# Arsenic exposure and lung fibrotic changes-evidence from a longitudinal cohort study and experimental models

**DOI:** 10.3389/fimmu.2023.1225348

**Published:** 2023-08-22

**Authors:** Chih-Wen Wang, Hsin-Ying Clair Chiou, Szu-Chia Chen, Da-Wei Wu, Hung-Hsun Lin, Huang-Chi Chen, Wei-Ting Liao, Ming-Hong Lin, Chih-Hsing Hung, Chao-Hung Kuo

**Affiliations:** ^1^ Division of Hepatobiliary, Department of Internal Medicine, Kaohsiung Medical University Hospital, Kaohsiung Medical University, Kaohsiung, Taiwan; ^2^ Department of Internal Medicine, Kaohsiung Municipal Siaogang Hospital, Kaohsiung Medical University, Kaohsiung, Taiwan; ^3^ Teaching and Research Center, Kaohsiung Municipal Siaogang Hospital, Kaohsiung Medical University, Kaohsiung, Taiwan; ^4^ Kaohsiung Medical University Hospital, Kaohsiung Medical University, Kaohsiung, Taiwan; ^5^ Department of Applied Chemistry, National Chi Nan University, Nantou, Taiwan; ^6^ Division of Nephrology, Department of Internal Medicine, Kaohsiung Medical University Hospital, Kaohsiung Medical University, Kaohsiung, Taiwan; ^7^ Research Center for Environmental Medicine, Kaohsiung Medical University, Kaohsiung, Taiwan; ^8^ Faculty of Medicine, College of Medicine, Kaohsiung Medical University, Kaohsiung, Taiwan; ^9^ Division of Pulmonary and Critical Care Medicine, Department of Internal Medicine, Kaohsiung Medical University Hospital, Kaohsiung Medical University, Kaohsiung, Taiwan; ^10^ Department of Biotechnology, College of Life Science, Kaohsiung Medical University, Kaohsiung, Taiwan; ^11^ Department of Medical Research, Kaohsiung Medical University Hospital, Kaohsiung Medical University, Kaohsiung, Taiwan; ^12^ Department of Microbiology and Immunology, School of Medicine, College of Medicine, Kaohsiung Medical University, Kaohsiung, Taiwan; ^13^ M.Sc. Program in Tropical Medicine, College of Medicine, Kaohsiung Medical University, Kaohsiung, Taiwan; ^14^ Department of Pediatrics, Kaohsiung Medical University Hospital, Kaohsiung Medical University, Kaohsiung, Taiwan; ^15^ Department of Pediatrics, Kaohsiung Municipal Siaogang Hospital, Kaohsiung Medical University, Kaohsiung, Taiwan; ^16^ Division of Gastroenterology, Department of Internal Medicine, Kaohsiung Medical University Hospital, Kaohsiung Medical University, Kaohsiung, Taiwan

**Keywords:** arsenic, lung fibrosis, epithelial-mesenchymal transition, apigenin, LDCT images

## Abstract

**Introduction:**

Arsenic (As) exposure is associated with lung toxicity and we aim to investigate the effects of arsenic exposure on lung fibrotic changes.

**Methods:**

Participants (n= 976) enrolled *via* a general health survey underwent chest low-dose computed tomography (LDCT), spirometry forced expiratory volume in 1 s (FEV1), forced vital capacity (FVC), and urinary arsenic examination during 2016 and 2018. Lung fibrotic changes from LDCT were defined. As^LtoL^, low arsenic levels in both 2016 and 2018; As^LtoH^, low arsenic in 2016 but high levels in 2018; As^HtoL^, high arsenic in 2016 but low levels in 2018; As^HtoH^, high arsenic levels in both 2016 and 2018. Mice exposed to 0. 0.2mg/L, 2 mg/L, 50 mg/L of sodium arsenite (NaAsO_2_) through drinking water for 12 weeks and 24 weeks were applied for histological analysis. Cultured lung epithelial cells were exposed to NaAsO_2_ and the mesenchymal changes were examined.

**Results:**

As^HtoH^ increased the risk (OR= 1.65, 95% CI 1.10, 2.49) of Lung fibrotic _positive to positive_ (reference: Lung fibrotic _negative to negative_) compared with As^LtoL^. Moreover, the predicted mean of FVC and FEV1 in As^HtoH^ (−0.09 units, 95% CI: −0.27, −0.09; −0.09 units, 95% CI: −0.17, −0.01) and As^LtoH^ (−0.13 units, 95% CI: −0.30, −0.10; −0.13 units, 95% CI: −0.22, −0.04) was significantly lower than AS^LtoL^. Significant lung fibrotic changes including the increase of the alveolar septum thickness and collagen fiber deposition were observed upon 2 mg/L NaAsO_2_ treatment for 12 weeks, and the damage was dose- and time-dependent. *In vitro*, sodium arsenite treatment promotes the epithelial-mesenchymal transition (EMT)-like changes of the normal human bronchial epithelial cells, including upregulation of several fibrotic and mesenchymal markers (fibronectin, MMP-2, and Snail) and cell migration. Inhibition of reactive oxygen species (ROS) and MMP-2 impaired the arsenic-induced EMT changes. Administration of a flavonoid, apigenin, inhibited EMT *in vitro* and pulmonary damages *in vivo* with the reduction of mesenchymal markers.

**Discussion:**

we demonstrated that continued exposure to arsenic causes lung fibrosis in humans and mice. Targeting lung epithelial cells EMT is effective on the development of therapeutic strategy. Apigenin is effective in the inhibition of arsenic-induced pulmonary fibrosis and EMT.

## 1 Introduction

Arsenic (As) is classified as a primary lung carcinogen (1) and is ranked as the top toxic substance on the Agency for Toxic Substances and Disease Registry’s priority list (2). An estimated 94-220 million people worldwide are at risk of being exposed to elevated levels of arsenic in groundwater, with the vast majority (94%) of those affected residing in Asia (3). Studies in epidemiology have indicated that exposure to arsenic is linked to heightened inflammation in the airways (4), and is inversely associated with forced expiratory volume in one second (FEV1) and forced vital capacity (FVC) in both the general population (5) and children (6). Among populations exposed to low levels of arsenic, prolonged consumption of contaminated drinking water has been linked to adverse respiratory effects in adult men who do not smoke (N=834; drinking water with arsenic concentration ranging from 11-50 μg/L) (7–9). A systematic review has shown that exposure to arsenic is inversely associated with FEV1 and FVC, but not with the ratio of FEV1 to FVC. This implies that arsenic exposure is a risk factor for restrictive lung disease (10). Residents living in close proximity to petrochemical complexes have been found to exhibit elevated levels of urinary arsenic, which can increase the likelihood of respiratory symptoms and illnesses. We hypothesize that such arsenic exposure increases the risk of chronic lung inflammation, which in turn contributes to the development of interstitial lung changes in individuals living near petrochemical complexes. Nevertheless, there is still a lack of image-based evidence and repeated measurements to demonstrate the impact of arsenic exposure on lung fibrotic changes.

Fibrosis is characterized by excess deposition of extracellular matrix. With repeated external stimuli, the damaged epithelial cells undergo wound healing through the activation of epithelial-mesenchymal transition (EMT) (11), which is characterized by increased cell mobility, cytoskeleton reorganization, increased invasiveness, and extracellular matrix expressions. EMT of pulmonary epithelial cell promote a pro-fibrotic microenvironment (12) and is believed as a possible therapeutic target for lung fibrosis (13). The activation of core EMT signaling, such as TGF-β, WNT, and hedgehog pathways, activates the differentiation of epithelial cells as well as fibrocytes, endothelial cells, and resident fibroblasts, into the fibrogenic myofibroblasts, thus increasing the extracellular matrix (ECM) deposition. Moreover, as a physical barrier, damage of the epithelial cells further activates immune responses through secretory factors. The activated immune cells, for example, Th2-M2 type cells, will further potentiate the fibrotic changes through promote EMT of epithelial cells and differentiation into myofibroblasts (14). Arsenic exposure was shown to be bi-directionally correlated with TGF-β/Smad signaling and lung fibrosis (15). More recently, by using animal models, exposure to arsenic (at least 2.5mg/kg/d for 4 months) was demonstrated to induce lung inflammation and fibrosis. The activation of HMGB1/RAGE, PI3K/AKT, and TGF-β signaling were detected in lung tissue (16). Mice exposed to arsenic lead to lung fibrosis (at least 5 ppm for 6 months) *via* stimulating the extracellular vehicles secretion from lung epithelial cells to activate myofibroblast (17). Similarly, mice exposed to arsenic through drinking water cause lung fibrosis (at least 10 ppm for 12 months) and upregulation of the M2 marker, Arg1, expressions in mice lung (18). Together these results demonstrated the causal relationship of arsenic exposure and lung fibrosis, and several involved mechanisms. Arsenic was long known as a carcinogen. The health impact evaluation of arsenic exposure regarding non-tumorigenic effects, such as lung fibrosis, was largely lacking. Following a repeated cross-sectional study with chest low-dose computed tomography (LDCT) images taken during 2016 and 2018, we sought to investigate and replicate a plausible link between lung fibrosis and exposure to arsenic by utilizing a mouse exposure mode.

Treatment of arsenic toxicity is still challenging because of the deleterious effects of chelating agents. Apigenin (4,5,7-trihydroxyflavone) is a naturally occurring flavone that is abundant in many vegetables and fruits, and it has been demonstrated to have pharmacological effects including anti-inflammatory, antioxidative, and antitumor (19). In a rodent disease model, apigenin ameliorated bleomycin-induced lung fibrosis by suppressing TNF-α and TGF-β levels, SOD activity, and hydroxyproline content (20). Moreover, apigenin can inhibit cancer-related type III EMT, thus suppressing the migration and invasion of cancer cells in hepatocellular carcinoma (21), colon cancer (22), and prostate cancer (23). Besides, some flavonoids were demonstrated to have chelating activities to reduce arsenic toxicity (24). EMT is considered a common pathological mechanism of several fibrotic lung diseases, such as idiopathic pulmonary fibrosis, chronic obstructive pulmonary disease, and asthma (25). Targeting the EMT process is believed an effective strategy to control fibrotic diseases (26–29). Here, we have applied *in vitro* cell culture model and *in vivo* mice model to evaluate the therapeutic efficacy and mechanisms of apigenin toward arsenic-induced lung fibrosis.

## 2 Materials and methods

### 2.1 Participants recruitment

Dalinpu is a region located in the Xiaogang District of southern Taiwan’s Kaohsiung City. It is encompassed by the Linhai and Linyuan petrochemical industrial parks, as well as an oil refinery park. These industrial facilities employ multiple methods, such as vacuum distillation, catalytic reforming, naphtha cracking, distillation, and hydrodesulfurization, to produce a range of products, including chemicals, lubricants, diesel, and gasoline. In 2016, we conducted a health survey among the general population in southern Taiwan, using advertisements to recruit individuals aged 20 years or above. The same participants were followed up in 2018. A total of 8 subjects were excluded from the analysis due to their history of lung carcinoma. Among these exclusions, 4 subjects belonged to the As^HtoH^ group, 2 belonged to the As^LtoH^ group, 1 belonged to the As^HtoL^ group, and 1 belonged to the As^LtoL^ group. Additionally, 1 subject with a history of lung tuberculosis and 4 subjects with a history of asthma were also excluded from the analysis. Among the subjects with asthma history, 2 belonged to the As^HtoH^ group, 1 belonged to the As^LtoH^ group, and 1 belonged to the As^LtoL^ group (**Table 1**). All study participants underwent lung function testing, LDCT, and urinary arsenic examination in 2016. They were then followed up and underwent the same tests in 2018. A total of 976 participants were included in the study (**Figure S1**). The participants were advised to avoid seafood consumption 3 days before the tests. The study protocol was approved by the Institutional Review Board of Kaohsiung Medical University Hospital (Number: KMUHIRB-G(II)-20190011). All participants provided informed consent before study enrollment.

**Table 1:** Demographic characteristics, lung function tests and lung fibrotic changes by urinary arsenic levels in 2016 and 2018 (n= 976).

Characteristics	All	As^LtoL*^	As^HtoL*^	As^LtoH*^	As^HtoH*^	p^‡^
	(n=976)	(n=394)	(n=167)	(n=159)	(n=256)	
Continuous variable, mean (SD)						
Age (yrs.)	56.9(11.0)	52.7(10.3)	58.0(10.7)	58.5(10.4)	61.7(10.4)	<0.001
BMI (Kg/m^2^)	25.1(3.9)	24.8(4.0)	25.4(4.2)	24.8(3.6)	25.4(3.7)	0.186
Lung function tests (L)						
2016 (visit 1)						
FVC	2.6(0.8)	2.8(0.8)	2.4(0.7)	2.6(0.8)	2.4(0.7)	<0.001
FEV1	2.3(0.7)	2.5(0.7)	2.2(0.6)	2.3(0.7)	2.1(0.6)	<0.001
2018 (visit 2)						
FVC	2.5(0.8)	2.6(0.8)	2.4(0.8)	2.4(0.7)	2.3(0.7)	<0.001
FEV1	2.3(0.7)	2.5(0.7)	2.2(0.7)	2.2(0.7)	2.1(0.7)	<0.001
Category variable, n (%)						
Gender						0.017
Female	618(63.3)	235(59.6)	118(70.7)	92(57.9)	173(67.6)	
Male	358(36.7)	159(40.4)	49(29.3)	67(42.1)	83(32.4)	
Educational level						<0.001
≤junior high school	481(49.3)	128(32.5)	89(53.3)	89(56.0)	175(68.4)	
Senior high school	323(33.1)	164(41.6)	50(29.9)	48(30.2)	61(23.8)	
≥college	172(17.6)	102(25.9)	28(16.8)	22(13.8)	20(7.8)	
Smoking						0.069
Yes	102(10.5)	50(12.7)	18(10.8)	18(11.3)	16(6.3)	
No	874(89.5)	344(87.3)	149(89.2)	141(88.7)	240(93.8)	
Alcohol consumption						0.464
Yes	182(18.6)	79(20.1)	25(15.0)	27(17.0)	51(19.9)	
No	794(81.4)	315(79.9)	142(85.0)	132(83.0)	205(80.1)	
Betel chewing						0.872
Yes	19(1.9)	7(1.8)	4(2.4)	4(2.5)	4(1.6)	
No	957(98.1)	387(98.2)	163(97.6)	155(97.5)	252(98.4)	
Air purifier						0.038
Yes	816(83.6)	326(82.7)	133(79.6)	129(81.1)	228(89.1)	
No	160(16.4)	68(17.3)	34(20.4)	30(18.9)	28(10.9)	
Diabetes mellitus						<0.001
Yes	318(32.6)	98(24.9)	59(35.3)	54(34.0)	107(41.8)	
No	658(67.4)	296(75.1)	108(64.7)	105(66.0)	149(58.2)	
Hypertension						<0.001
Yes	591(60.6)	200(50.8)	105(62.9)	102(64.2)	184(71.9)	
No	385(39.4)	194(49.2)	62(37.1)	57(35.8)	72(28.1)	
Pairwise lung fibrotic^†^						0.004
Negative to negative	555(56.9)	252(64.0)	95(56.9)	81(50.9)	127(49.6)	
Positive to negative	116 (11.9)	44 (11.2)	18 (10.8)	21 (13.2)	33 (12.9)	
Negative to positive	66 (6.8)	25 (6.3)	10 (6.0)	17 (10.7)	14 (5.5)	
Positive to positive	239 (24.5)	73 (18.5)	44 (26.3)	40 (25.2)	82 (32.0)	
Excluding subjects						
Lung carcinoma, n (%)	8 (100.0)	1 (12.5)	1 (12.5)	2 (25.0)	4 (50.0)	0.294
Lung tuberculosis, n (%)	1 (100.0)	0 (0)	0 (0)	1 (100.0)	0 (0)	NA
Asthma history	4 (100.0)	1 (25.0)	0 (0)	1 (25.0)	2 (50.0)	NA

*Pairwise urinary arsenic changes: urinary arsenic levels (μg/g creatinine) were categorized into high (H: ≥100) and low (L: <100). As^LtoL^: arsenic level low in 2016 and low in 2018. As^LtoH^: arsenic level low in 2016 and high in 2018. As^HtoL^: arsenic level high in 2016 and low in 2018. As^HtoH^: arsenic level high in 2016 and high in 2018. †Pairwise lung fibrotic changes: “Lung fibrotic _negative to negative_” indicating absence of lung fibrotic changes in both 2016 and 2018, “Lung fibrotic _negative to positive_” indicating no lung fibrotic changes in 2016 but present in 2018, “Lung fibrotic _positive to negative_” indicating lung fibrotic changes in 2016 but not in 2018, and “Lung fibrotic _positive to positive_” indicating lung fibrotic changes present in both 2016 and 2018. ‡Chi-square and ANOVA test in As^LtoL^, As^LtoH^, As^HtoL^, As^HtoH^. As, arsenic

### 2.2 Urinary arsenic, spirometry measurements, and CT image acquisition

To determine the concentration of arsenic in the urinary samples, nitric acid was used to digest the samples. Inductively coupled plasma-mass spectrometry (ICP-MS), specifically the Thermo Scientific X-SERIES II instrument from Germany, was employed for the analysis. The method for measuring arsenic levels using ICP-MS has been previously described in other studies (30). The monoisotopic mass of arsenic was determined to be 75. To ensure experimental accuracy, standard reference materials for human urine, specifically the Seronorm™ Trace Elements Urine (ref. SERO AS, Billingstad, Norway), were tested. The average recovery rate for the urinary arsenic content was found to be 95%, with a detection limit of 0.03 μg/L. To account for urine dilution, the concentrations were adjusted for urinary creatinine and reported as μg/g creatinine.

Spirometry testing was carried out in accordance with the 2005 technical standards set by the American Thoracic Society and the European Respiratory Society (31).

LDCT scans were conducted using thin-slice scanners (Toshiba Aquilion One 640 slices CT, Japan) equipped with 16 cm PUREViSION detectors. The scans were performed without contrast enhancement and covered the lung area from apex to base. All scans were obtained using a low-dose regimen, with the machine set at 120 kVp, 9 (15 mA/0.6 s) or 21 (35 mA/0.6 s) mAs, 1.5:1 pitch ratio, and 0.35 seconds rotation time. The effective radiation dose ranged from 0.3 mSv to 0.8 mSv. Lung fibrotic changes were defined as the presence of curvilinear or linear densities, fine lines, or plate opacity in specific lobes on LDCT images. The observed radiological results can suggest previous lung inflammation leading to scarring or fibrosis of lung tissue. As a result, these changes may lead to a decrease in lung function and impaired respiratory capacity.

### 2.3 Animals and lung histology, immunohistochemistry

Animals Use Protocol (IACUC-109251) was approved by the Kaohsiung Medical University-Institutional Animal Care and Use Committee. C57BL/6 mice aged 6–8 weeks were purchased from the National Laboratory Animal Center (Taiwan) and were housed in an Association for Assessment and Accreditation of Laboratory Animal Care International (AAALAC)-accredited facility of Kaohsiung Medical University. After 2 weeks of adaptation, mice were randomly grouped into 6 mice per group. Mice were exposed to 0, 0.2, 2, and 50 mg/L of NaAsO_2_ (Sigma, St. Louis, MO, purity: 99.0%) through drinking water for 12 and 24 weeks. For evaluation of the therapeutic effects of apigenin, mice were divided into 4 groups with 6 mice per group including control, 50mg/L NaAsO_2_, 50mg/L NaAsO_2_ combined with 10mg/kg/day of apigenin, and 10mg/kg/day of apigenin. The control group was reserved without any treatment.

After treatment, the mice were sacrificed by anesthesia with CO_2_ and perfused with phosphate-buffered saline. Mice left lungs were perfused and fixed with 10% formalin and embedded in paraffin. Sections in 3-µm thickness were placed on slides and applied for hematoxylin and eosin (H&E), Masson’s trichrome staining, and immunohistochemistry staining. Immunohistochemistry was performed using UltraVision Quanto Detection System HRP DAB (Thermo, TL-060-QHD). Briefly, the deparaffinized and rehydrated section were covered using Hydrogen Peroxide Block (Thermo, TL-060-QHD). Antigen retrieval was performed using Tris-EDTA (pH9.0) buffer and followed by immunoblot for MMP-2 (Genetex, GTX104577), the antibody was diluted 1:500 in PBS and incubated at room temperature for 2 hrs. For Snail (Genetex, GTX100754, 1:400), fibronectin (Genetex 112794, 1:200), and COL1A1 (Cell signaling #72026, 1:200) the antibody was diluted in PBS and incubated at 4°C overnight. After three times washes with PBS, sections were then incubated with a secondary antibody followed by DAB chromogen. The sections were counterstained with hematoxylin and mounted with a xylene-based mounting medium. The images were scanned using the Olympus VS200 slide scanner (Olympus, Japan) and captured and analyzed using Olyvia software. Masson’s trichrome staining was performed according to the manufacturer’s protocol (Sigma-Aldrich, USA)

### 2.4 Cell culture, drugs, and wound healing assay

Normal human bronchial epithelial cells (NHBE, Lonza) were maintained in Keratinocyte SFM basal medium supplemented with 5ug/L human recombinant epithelial growth factor (EGF), 50mg/L Bovine Pituitary Extract (BPE), 5mg/L insulin and 25nM hydrocortisone at 37°C in a 5% CO2 atmosphere. Drugs including NaAsO_2_ (#S7400), N-acetylcysteine (NAC, #A7250), and apigenin (#10798), were purchased from Sigma-Aldrich (St. Louis, MO, USA). ARP-100 was purchased from Santa Cruz (Santa Cruz, CA).

NHBE cells were plated in 12 well plates and let to grow to confluence. Inhibitors were pre-treated 2h before Arsenic treatment. Wounds were made with 200ul tips, and PBS wash for two times to remove the floating cells. NaAsO_2_ was added in the presence of inhibitors and incubated for 12 hours. The images were captured using microscopy and the wound area was analyzed by Image J. The wound area was normalized with 0 hours as 100% and expressed as GAP%.

### 2.5 Western blotting

After arsenic treatments for indicated times, the cells were washed twice with ice-cold PBS, and lysed immediately by adding the lysis buffer directly into the culture plate. The composition of lysis buffer is 50mM Tris-HCl, 150mM Sodium chloride, 1% NP-40, 0.5% Sodium deoxycholate, 0.1% SDS, 5mM EDTA, protease inhibitor (cOmplete™, Mini, EDTA-free Protease Inhibitor Cocktail, Sigma-Aldrich, USA) and phosphatase inhibitor (PhosStop™, Sigma-Aldrich, USA). The protein lysate was harvested by centrifugation with 14,000 rpm at 4°Cfor 15 min to pellet the cell debris. The protein concentration was determined using BCA Dual-Range BCA Protein Assay Kit (Visual Protein, Taiwan). A total of 10 ug of protein lysate was separated in 6~13% SDS-PAGE and transferred onto PVDF membranes (Millipore, USA). After blocking at 5% skim milk at room temperature for 1 hour, the membrane was incubated with the first antibody at 4°C overnight. After three times washes with 0.1%TBST, the membrane was incubated with Horseradish Peroxidase (HRP)-conjugated anti-rabbit IgG or anti-goat secondary antibody respectively (Jackson ImmunoResearch Laboratories, West Grove, PA). The membrane was developed by reacting with chemiluminescence HRP substrate (Immobilon^®^ Western, EMD Millipore Corporation, Burlington, MA, Germany) and the protein bands were visualized and captured using ChemiDoc-It 810 Imager (Ultra-Violet Products, USA). The protein bands were quantified using Image J. Antibodies used are including MMP-2 (1:1,000; Genetex, GTX104577, Xinzhu, Taiwan), Fibronectin (1:10,000; F3648, Sigma, St. Louis, MO), GAPDH (1:20,000; GeneTex, GTX100118, Xinzhu, Taiwan), and Snail (1:1,000; GeneTex, GTX100754, Xinzhu, Taiwan), goat-anti-rabbit IgG (HRP) (Jackson ImmunoResearch Laboratories inc.). All experiments were performed at least three times.

### 2.6 Immunofluorescence

NHBE cells were treated with 10μM of apigenin for 2 hours followed by combined treatment with 1μM NaAsO_2_ for additional 24 hours. After treatment, the cells were fixed with 10% formalin at room temperature for 30 min. After washing three times with PBS, the cells were then permeabilized with 0.1% Triton X-100 in PBS for 5 min. Followed by 3 times of PBS wash, the cells were incubated with anti-Fibronectin antibody (1:500, F3648, Sigma, St. Louis, MO) at 4°C overnight. The non-specific binding was washed with 0.02% Tween-20 in PBS (0.02% PBST), followed by incubation with goat-anti-rabbit-Alexa488 (Jackson ImmunoResearch Laboratories Inc.) at room temperature for 2 hours. After PBS wash, the cells were mounted using mounting solution with DAPI. The cells were analyzed by laser confocal microscope (FV1000, OLYMPUS IX-81).

### 2.7 Statistical analysis

Urinary arsenic levels (measured in µg/g creatinine) obtained in 2016 and 2018 were classified based on the guidelines of the Agency for Toxic Substances and Disease Registry (ATSDR) into two groups: high (H; ≥100 µg/g creatinine) and low (L; <100 µg/g creatinine) (2). The modifications in urinary arsenic concentrations between 2016 and 2018 were classified into four categories: (1) As^LtoL^, indicating low arsenic levels in both 2016 and 2018; (2) As^LtoH^, indicating low arsenic levels in 2016 but high levels in 2018; (3) As^HtoL^, indicating high arsenic levels in 2016 but low levels in 2018; and (4) As^HtoH^, indicating high arsenic levels in both 2016 and 2018. We analyzed the association between urinary arsenic levels (logarithmic transformation) and lung function tests by using multiple linear regression analysis adjusted for age, gender, BMI, active smoking, educational levels, hypertension, and diabetes mellitus history. Linear mixed models were utilized to examine the link between changes in urinary arsenic levels and lung function tests. A first-order autoregressive error structure was accounted for within-patient correlation. To analyze the differences in lung function test changes, we added the interaction of time with urinary arsenic changes to the model. Variables that were (1) associated with outcomes, (2) associated with the exposure of interest and (3) based on relevant literature were selected for the analysis. For the model analysis, age, sex, hypertension, diabetes mellitus, educational level, and air purifier uses which showed significant association with urinary arsenic changes, were selected. Additionally, body mass index (BMI) and smoking which were associated with lung function tests in previous studies were included in the model analysis. Finally, we identified potential confounding variables, such as age, sex, hypertension, diabetes mellitus, educational level, air purifier use, and smoking, and adjusted for them in the model. Linear mixed models were used, accounting for within-patient correlation by incorporating a first-order autoregressive error structure.

Based on the chest LDCT scans in 2016 and 2018, participants were classified as having either negative or positive lung fibrotic changes. The LDCT findings were classified as follows: “Lung fibrotic _negative to negative_” indicating the absence of lung fibrotic changes in both 2016 and 2018, “Lung fibrotic _negative to positive_” indicating no lung fibrotic changes in 2016 but present in 2018, “Lung fibrotic _positive to negative_” indicating lung fibrotic changes in 2016 but not in 2018, and “Lung fibrotic _positive to positive_” indicating lung fibrotic changes present in both 2016 and 2018. To determine the relationship between changes in urinary arsenic levels and the results of lung fibrotic changes, a multinomial logistic regression was performed, with “lung fibrotic _negative to negative_” being used as the reference category. Cellular protein expression data were analyzed using a one-way analysis of variance followed by Dunnett’s multiple comparison test. All analyses were performed using SPSS software version 22 (IBM Corp., Armonk, NY, USA). The significance level was set at p < 0.05.

## 3 Results

### 3.1 Demographic characteristics

In 2016 and 2018, 423 (43.3%) and 416 (42.6%) of the participants, respectively, had high levels of urinary arsenic (≥100 ug-g/creatinine). The presence of lung fibrotic changes was observed in 355 (36.4%) and 305 (31.3%) of the participants in 2016 and 2018, respectively. Compared to the other groups, the As^HtoH^ group had participants who had significantly older age (yrs.) (As^HtoH^: 61.7 vs. As^LtoH^: 58.5, vs. As^HtoL^: 58.0, vs. As^LtoL^: 52.7; p< 0.001). Additionally, this group had a higher proportion of participants with diabetes mellitus (41.8% vs. 34.0% for As^LtoH^, 35.3% for As^HtoL^, and 24.9% for As^LtoL^; p < 0.001), a higher proportion of participants with hypertension (71.9% vs. 64.2% for As^LtoH^, 62.9% for As^HtoL^, and 50.8% for As^LtoL^; p < 0.001), and a higher proportion of participants with Lung fibrotic _positive to positive_ (32.0% vs. 25.2% for As^LtoH^, 26.3% for As^HtoL^, and 18.5% for As^LtoL^; p < 0.001) (**Table 1**). In addition, the As^LtoL^ group had participants with a significantly higher proportion of college educational level (25.9% vs. 16.8% for As^HtoL^, 13.8% for As^LtoH^, and 7.8% for As^HtoH^; p < 0.001). The As^HtoL^ group had participants with a significantly higher proportion of female gender (70.7% vs. 59.6% for As^LtoL^, 57.9% for As^LtoH^, and 67.6% for As^HtoH^; p= 0.017). Interestingly, the As^HtoH^ group had participants with a significantly higher proportion of air purifier use (89.1% vs. 82.7% for As^LtoL^, 79.6% for As^HtoL^, and 81.1% for As^LtoH^; p= 0.017). The changes in urinary arsenic levels did not show any significant difference with respect to BMI, smoking, alcohol consumption, and betel chewing, as shown in **Table 1**. Participants with As^HtoH^ had significantly lower FVC (L) (As^HtoH^: 2.4 vs. As^LtoH^: 2.6, As^HtoL^: 2.4, As^LtoL^: 2.8, p< 0.001 in 2016; As^HtoH^: 2.3 vs. As^LtoH^: 2.4, As^HtoL^: 2.4, As^LtoL^: 2.6, p< 0.001 in 2018) and FEV1 (L) (As^HtoH^: 2.1 vs. As^LtoH^: 2.3, As^HtoL^: 2.2, As^LtoL^: 2.5, p< 0.001 in 2016; As^HtoH^: 2.1 vs. As^LtoH^: 2.2, As^HtoL^: 2.2, As^LtoL^: 2.5, p< 0.001 in 2018) than As^LtoL^, As^LtoH^and As^HtoL^ (**Table 1**). Participants with Lung fibrotic_positive to positive_ had significantly higher urinary arsenic levels (µg/g creatinine) in 2016 (geometric mean: Lung fibrotic_positive to positive_: 110.1 vs. Lung fibrotic_negative to positive_: 81.7 vs. Lung fibrotic_positive to negative_: 90.9 vs. Lung fibrotic_negative to negative_: 86.0, p= 0.001) and 2018 (geometric mean: Lung fibrotic_positive to positive_: 94.9 vs. Lung fibrotic_negative to positive_: 89.8 vs. Lung fibrotic_positive to negative_: 91.2 vs. Lung fibrotic_negative to negative_: 80.9, p= 0.040) than Lung fibrotic_negative to positive_, Lung fibrotic_positive to negative_ and Lung fibrotic_negative to negative_ (**Table S1**)

### 3.2 Association between lung function tests and changes in urinary arsenic levels

The mean FVC (L), and FEV1 (L) were 2.45, 2.28 in 2018 and 2.58, and 2.33 in 2016 respectively (**Table S2**). We utilized multiple linear regression analysis while adjusting for various factors such as age, gender, BMI, active smoking, educational levels, hypertension, and diabetes mellitus history. Our findings revealed significant negative associations between urinary arsenic levels (after logarithmic transformation) and two key measures of lung function: FVC and FEV1. In the year 2016, we observed a significant negative association between urinary arsenic levels (logarithm transformed) and FVC (β = -0.199, 95% CI -0.210 to -0.029, p = 0.010). Similarly, in 2018, we found a significant negative association between urinary arsenic levels and FVC (β = -0.118, 95% CI -0.216 to -0.020, p = 0.010). Furthermore, we observed a significant negative association between urinary arsenic levels and FEV1 in 2016 (β = -0.111, 95% CI -0.190 to -0.033, p = 0.006). Similarly, in 2018, we found a significant negative association between urinary arsenic levels and FEV1 (β = -0.120, 95% CI -0.211 to -0.029, p = 0.010) (**Table S3**).

In linear mixed models, we found that the predicted mean of FVC and FEV1 in As^HtoH^ (−0.40 units, 95% CI: −0.52, −0.28, p< 0.001; −0.39 units, 95% CI: −0.50, −0.28, p< 0.001), As^LtoH^ (−0.26 units, 95% CI: −0.39, −0.12, p< 0.001; −0.25 units, 95% CI: −0.37, −0.12, p< 0.001) and As^HtoL^ (−0.29 units, 95% CI: −0.42, −0.15, p< 0.001; −0.29 units, 95% CI: −0.41, −0.16, p< 0.001) was significantly lower than AS^LtoL^ in crude analysis (**Table 2**). After adjustment for age, gender, BMI, smoking, education, air purifier use, hypertension, and diabetes mellitus (model 2), we found that the predicted mean of FVC and FEV1 in As^HtoH^ (−0.09 units, 95% CI: −0.27, −0.09, p = 0.046; −0.09 units, 95% CI: −0.17, −0.01, p = 0.024) and As^LtoH^ (−0.13 units, 95% CI: −0.30, −0.10, p = 0.008; −0.13 units, 95% CI: −0.22, −0.04, p = 0.006) was significantly lower than AS^LtoL^ (**Table 2**).

**Table 2:** Association between pairwise urinary arsenic changes and lung function tests in participants during 2016 and 2018 determined using a linear mixed model (n= 976).

Items	FVC			FEV1		
	β	95%CI	p	β	95%CI	p
Crude						
Pairwise Arsenic changes						
As^HtoH^	-0.4	(-0.52,-0.28)	<0.001	-0.39	(-0.50,-0.28)	<0.001
As^LtoH^	-0.26	(-0.39,-0.12)	<0.001	-0.25	(-0.37,-0.12)	<0.001
As^HtoL^	-0.29	(-0.42,-0.15)	<0.001	-0.29	(-0.41,-0.16)	<0.001
As^LtoL^	Ref			Ref		
Time						
2016	0.11	(0.09,0.17)	<0.001	0.05	(0.02,0.09)	0.001
2018	Ref			Ref		
Interaction: Time by pairwise Arsenic changes						
2016 by As^HtoH^	-0.02	(-0.08,0.05)	0.582	-0.01	(-0.06,0.04)	0.632
2016 by As^LtoH^	0.09	(0.02,0.17)	0.015	0.07	(0.01,0.14)	0.017
2016 by As^HtoL^	-0.08	(-0.16,-0.01)	0.027	-0.06	(-0.12,0.003)	0.064
2016 by As^LtoL^	Ref			Ref		
Model 1^*^						
Pairwise Arsenic changes						
As^HtoH^	-0.18	(-0.27,-0.09)	<0.001	-0.18	(-0.27,-0.10)	<0.001
As^LtoH^	-0.2	(-0.30,-0.10)	<0.001	-0.19	(-0.29,-0.10)	<0.001
As^HtoL^	-0.09	(-0.19,0.01)	0.066	-0.11	(-0.20,-0.02)	0.021
As^LtoL^	Ref			Ref		
Time						
2016	0.11	(0.06,0.15)	<0.001	0.03	(-0.001,0.07)	0.059
2018	Ref			Ref		
Interaction: Time by pairwise Arsenic changes						
2016 by As^HtoH^	-0.03	(-0.09,0.04)	0.442	-0.02	(-0.07,0.03)	0.456
2016 by As^LtoH^	0.09	(0.01,0.17)	0.022	0.07	(0.01,0.14)	0.024
2016 by As^HtoL^	-0.11	(-0.18,-0.03)	0.006	-0.08	(-0.14,-0.02)	0.013
2016 by As^LtoL^	Ref			Ref		
Model 2^†^						
Pairwise Arsenic changes						
As^HtoH^	-0.09	(-0.27,-0.09)	0.046	-0.09	(-0.17,-0.01)	0.024
As^LtoH^	-0.13	(-0.30,-0.10)	0.008	-0.13	(-0.22,-0.04)	0.006
As^HtoL^	-0.05	(-0.19,0.01)	0.349	-0.06	(-0.15,0.03)	0.174
As^LtoL^	Ref			Ref		
Time						
2016	0.11	(0.06,0.15)	<0.001	0.03	(-0.002,0.07)	0.063
2018	Ref			Ref		
Interaction: Time by pairwise Arsenic changes						
2016 by As^HtoH^	-0.02	(-0.08,0.05)	0.614	-0.01	(-0.07,0.04)	0.648
2016 by As^LtoH^	0.09	(0.02,0.17)	0.017	0.08	(0.01,0.14)	0.018
2016 by As^HtoL^	-0.1	(-0.17,-0.02)	0.009	-0.07	(-0.13,-0.01)	0.02
2016 by As^LtoL^	Ref			Ref		

^*^Model 1 adjusted for age, gender, and BMI. ^†^Model 2 adjusted for age, gender, BMI, smoking, education, air purifier, hypertension, and diabetes mellitus

The mean FVC and FEV1 in 2016 were significantly higher than values in 2018 (FVC: 0.11 units, 95% CI 0.09, 0.17, p< 0.001; FEV1: 0.05 units, 95% CI 0.02, 0.09, p< 0.001) in crude analysis. However, the significant difference became borderline for FEV1 after the adjustment of confounding factors in model 2 (**Table 2**).

**Figure 1:**
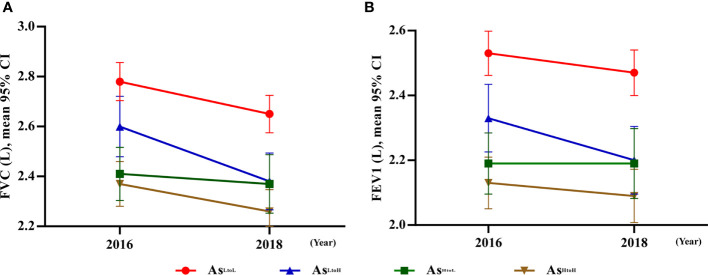
Significant interaction between urinary arsenic changes by time associated with lung function tests. **(A)** FVC: The difference in slop between As^LtoH^ and As^HtoL^, except As^HtoH^ was significantly different from 2016 to 2018 compared with As^LtoL^. **(B)** FEV1: The difference in slop between As^LtoH^ and As^HtoL^ except As^HtoH^ was significantly different from 2016 to 2018 compared with As^LtoL^. Abbreviation: As, arsenic.

The interaction between time and levels of arsenic changes was shown in **Table 2**. In crude analysis, the difference in slope of FVC changes in As^LtoH^ (β= 0.09; 95% CI 0.02, 0.17, p= 0.015) and As^HtoL^ (β= -0.08; 95% CI -0.16, -0.01, p= 0.027) was significantly different from As^LtoL^ (**Table 2**). In addition, the difference in slope of FEV1 changes in As^LtoH^ (β= 0.07; 95% CI 0.01, 0.14, p= 0.017) was significantly different from As^LtoL^. In model 2, the difference in slope of FVC changes in As^LtoH^ (β= 0.09; 95% CI 0.02, 0.17, p= 0.017) and As^HtoL^ (β= -0.10; 95% CI -0.17, -0.02, p= 0.009) was significantly different from As^LtoL^ (**Table 2**). In addition, the difference in slope of FEV1 changes in As^LtoH^ (β= 0.08; 95% CI 0.01, 0.14, p= 0.018) and As^HtoL^ (β= -0.07; 95% CI -0.13, -0.01, p= 0.020) was significantly different from As^LtoL^, which means that lung function changes are influenced by arsenic changes (**Figure 1**). The significant trend of the difference in slope of FVC and FEV1 changes was consistent in model 1 and model 2.

**Figure 2:**
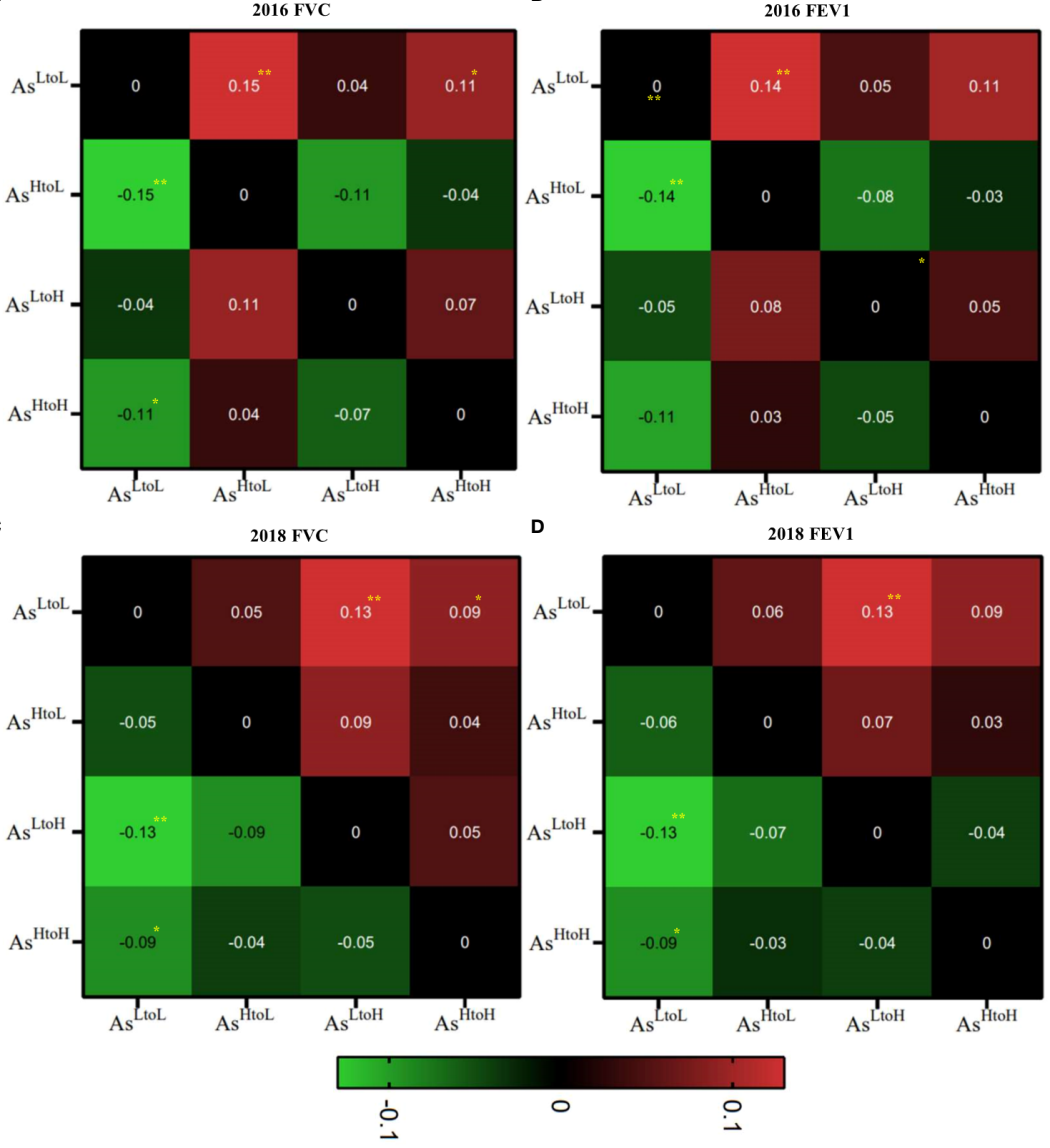
Heatmap of the mean differences in lung function tests, specifically FVC and FEV1, in relation to arsenic level changes over time were shown in each space. **(A)** FVC, **(B)** FEV1 in 2016 and **(C)** FVC, **(D)** FEV1 in 2018. We compared the lung function changes associated with arsenic changes (As^HtoH^, As^LtoH^, As^HtoL^ and As^LtoL^ in Y axis versus As^HtoH^, As^LtoH^, As^HtoL^ and As^LtoL^ in X axis). The mean differences in lung function tests were plotted on the Y-axis of arsenic changes against the corresponding arsenic changes patterns on the X-axis for comparative analysis. ^**^P< 0.01, ^*^P<0.05.

The mean difference of lung function tests associated with arsenic levels by time was shown in (**Figure 2** and **Table S4**). As^HtoH^, As^LtoH,^ and As^LtoL^ had significantly decreased FVC in 2018 compared with values in 2016 (mean difference, As^HtoH^: -0.09, 95% CI: -0.14, -0.04, p= 0.001; As^LtoH^: -0.20, 95% CI: -0.26, -0.14, p< 0.001; As^LtoL^: -0.11, 95% CI: -0.15, -0.06, p< 0.001), respectively. However, As^HtoL^ had no significant difference in FVC changes between 2016 and 2018 (**Figure 2** and **Table S4**). As^LtoH^ had significantly decreased FEV1 in 2018 compared with values in 2016 (mean difference, -0.11, 95% CI: -0.16, -0.05, p< 0.001) (**Figure 2** and **Table S4**). However, no significant difference in FEV1 changes between 2016 and 2018 in As^HtoH^, As^HtoL,^ and As^LtoL^ (**Figure 2** and **Table S4**). In 2016, As^LtoL^ has significantly higher FVC (vs. As^HtoH^ mean difference = 0.11; 95% CI: 0.02, 0.19, p = 0.015; vs. As^HtoL^ mean difference = 0.15; 95% CI: 0.05, 0.24, p = 0.003) and FEV1 (vs. As^HtoH^ mean difference = 0.11; 95% CI: 0.03, 0.18, p = 0.006; vs. As^HtoL^ mean difference = 0.14; 95% CI: 0.05, 0.22, p = 0.001) compared with As^HtoH^ and As^HtoL^. In 2016, there was no significant difference in FVC and FEV1 between As^HtoH^, As^LtoH,^ and As^HtoL^. In 2018, As^LtoL^ has significantly higher FVC (vs. As^HtoH^ mean difference = 0.09; 95% CI: 0.002, 0.18, p = 0.046; vs. As^LtoH^ mean difference = 0.13; 95% CI: 0.04, 0.23, p = 0.008) and FEV1 (vs. As^HtoH^ mean difference = 0.09; 95% CI: 0.01, 0.17, p = 0.024; vs. As^LtoH^ mean difference = 0.13; 95% CI: 0.04, 0.22, p = 0.006) compared with As^HtoH^ and As^LtoH^. In 2018, there was no significant difference in FVC and FEV1 between As^HtoH^, As^LtoH,^ and As^HtoL^ (**Figure 2** and **Table S4**).

### 3.3 Association between lung fibrotic changes and arsenic exposure

In the crude model, AS^HtoH^ (OR = 2.23; 95% CI: 1.52, 3.26; p< 0.001), AS^LtoH^ (OR = 1.71; 95% CI: 1.08, 2.70; p = 0.023) and AS^HtoL^ (OR = 1.60; 95% CI: 1.03, 2.49; p = 0.038) significantly increased the risk of lung fibrotic _positive to positive_ compared with AS^LtoL^ (using Lung fibrotic _negative to negative_ as the reference category) (**Table 3**). Moreover, AS^LtoH^ (OR = 2.12; 95% CI: 1.09, 4.11; p = 0.027) significantly increased the risk of lung fibrotic _negative to positive_ compared with AS^LtoL^ (using Lung fibrotic _negative to negative_ as the reference category) (**Table 3**). After adjustment for age, gender, BMI (model 1), we found that As^HtoH^ (OR = 1.77; 95% CI: 1.19, 2.64; p= 0.005) significantly increased the risk of lung fibrotic _positive to positive_ compared with AS^LtoL^ (using Lung fibrotic _negative to negative_ as the reference category). Additionally, As^LtoH^ (OR = 2.20; 95% CI: 1.12, 4.31; p= 0.021) significantly increased the risk of lung fibrotic _negative to positive_ compared with AS^LtoL^ (using Lung fibrotic _negative to negative_ as the reference category) (**Table 3**). After adjustment for age, gender, BMI, smoking, education, air purifier use, hypertension, and diabetes mellitus (model 2), we found that As^HtoH^ (OR = 1.65; 95% CI: 1.10, 2.49; p= 0.016) significantly increased the risk of lung fibrotic _positive to positive_ compared with AS^LtoL^ (using Lung fibrotic _negative to negative_ as the reference category). Additionally, As^LtoH^ (OR = 2.31; 95% CI: 1.16, 4.59; p= 0.017) significantly increased the risk of lung fibrotic _negative to positive_ compared with AS^LtoL^ (using Lung fibrotic _negative to negative_ as the reference category) (**Table 3**).

**Table 3:** Multinomial logistic regression analysis for lung fibrotic changes associated with urinary arsenic exposure (n= 976).

Arsenic changes	Lung fibrotic^‡^			Lung fibrotic^‡^			Lung fibrotic^‡^		
	negative to positive			positive to positive			positive to negative		
	OR	95% CI	p	OR	95% CI	p	OR	95% CI	p
Crude									
AS^HtoH^	1.11	(0.56,2.21)	0.764	2.23	(1.52,3.26)	<0.001	1.49	(0.90,2.45)	0.119
AS^LtoH^	2.12	(1.09,4.11)	0.027	1.71	(1.08,2.70)	0.023	1.49	(0.83,2.64)	0.179
AS^HtoL^	1.06	(0.49,2.29)	0.88	1.6	(1.03,2.49)	0.038	1.09	(0.60,1.97)	0.788
AS^LtoL^	Ref			Ref			Ref		
Model 1^*^									
AS^HtoH^	1.17	(0.58,2.37)	0.667	1.77	(1.19,2.64)	0.005	1.34	(0.80,2.24)	0.269
AS^LtoH^	2.2	(1.12,4.31)	0.021	1.47	(0.92,2.36)	0.107	1.41	(0.79,2.53)	0.251
AS^HtoL^	1.08	(0.50,2.35)	0.846	1.45	(0.92,2.27)	0.109	1.03	(0.56,1.89)	0.918
AS^LtoL^	Ref			Ref			Ref		
Model 2^†^									
AS^HtoH^	1.14	(0.55,2.35)	0.729	1.65	(1.10,2.49)	0.016	1.36	(0.80,2.32)	0.251
AS^LtoH^	2.31	(1.16,4.59)	0.017	1.43	(0.89,2.30)	0.14	1.44	(0.80,2.61)	0.226
AS^HtoL^	1.11	(0.51,2.42)	0.802	1.43	(0.90,2.26)	0.128	1.07	(0.58,1.96)	0.835
AS^LtoL^	Ref			Ref			Ref		

^*^Model 1 adjusted for age, gender, BMI. ^†^Model 2 adjusted for age, gender, BMI, smoking, education, air purifier, hypertension, and diabetes mellitus. ^‡^Reference: Lung fibrotic _negative to negative_ As, arsenic

### 3.4 Effect of arsenic exposure on lung pathology of mice

**Figure 3:**
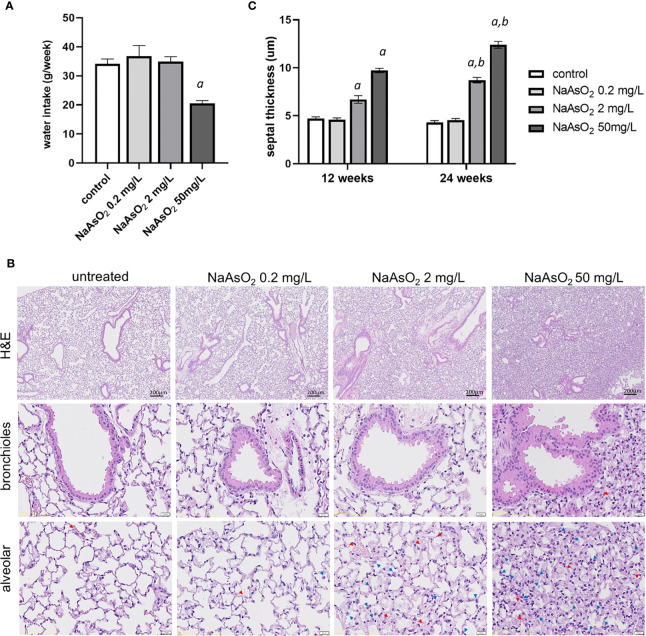
Effects of arsenic exposure on lung histology. **(A)**C57BL/6 mice aged 6-8 weeks were exposed to NaAsO_2_ at 0, 0.2mg/L, 2mg/L, 50mg/L for 24 weeks, and the **(A)** weekly water intake, **(B)** H&E stain after 24 weeks of arsenic exposure, and **(C)** alveolar septal thickness at 12 weeks and 24 weeks. Data was expressed as Mean+/- SEM. a: *p*<0.05 compared with control. b: *p*<0.05 compared with the same concentration at 12 weeks. Red arrow: red blood cell, blue arrow: immune cells.

To determine the dosage effects of arsenic exposure on lung fibrosis, mice were exposed to 0, 0.2 mg/L (low), 2mg/L (medium), and 50 mg/L (high) of NaAsO_2_ through drinking water for 24 weeks. The water intake was significantly lower in NaAsO_2_ 50 mg/L group compared with control (**Figure 3A**). There were no significant differences in body weight after 24 weeks of treatment compared with the control group. The exposure of low, medium, and high doses of NaAsO_2_ equal to approximately 0.03, 0.3, and 5 mg/kg body weight/day, respectively. Mice were sacrificed after 12 weeks and 24 weeks of exposure for histology analysis. Increased pulmonary hemorrhage (red arrow) and immune cells including macrophages and segmented neutrophil (blue arrow) were observed in mice treated with medium and high doses of NaAsO_2_ at 12 and 24 weeks. The representative images of 24 weeks of NaAsO_2_ exposure were shown (**Figure 3B**). The alveolar septal thickness was significantly increased after medium and high doses of NaAsO_2_ treatment for 12 weeks compared with the non-exposure control group. Similar results were observed at 24 weeks, which was significantly increased compared with that of 12 weeks (**Figures 3B, C**).

**Figure 4:**
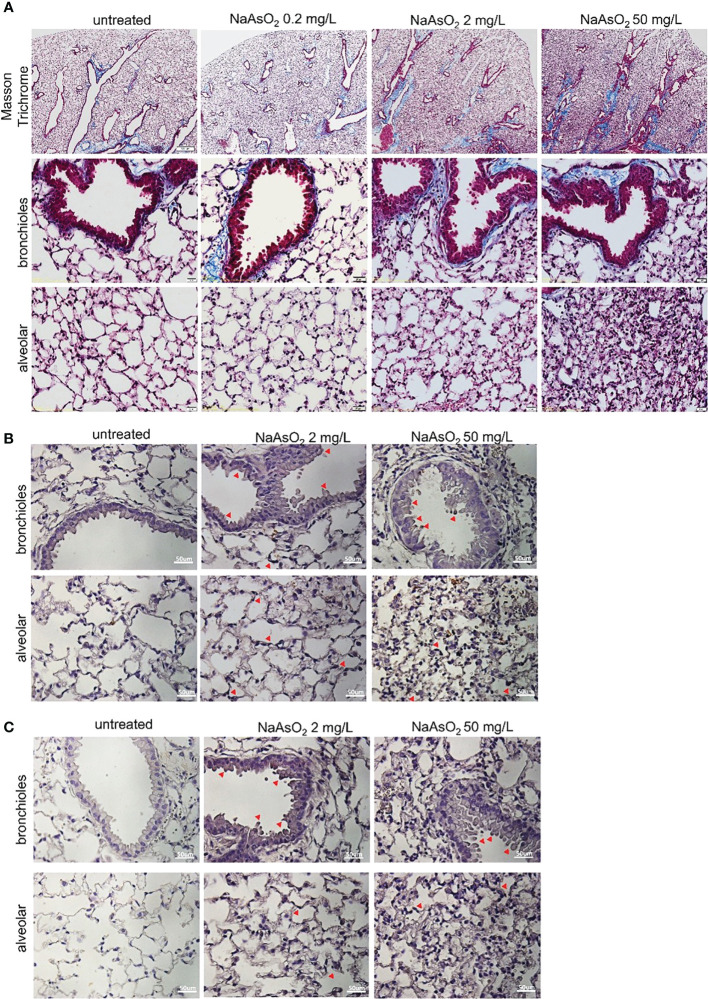
Effect of arsenic exposure on lung fibrosis C57BL/6 mice aged 6-8 weeks were exposed to NaAsO_2_ at 0, 2mg/L, 50mg/L for 24 weeks. **(A)** To show fibrosis in the bronchioles and alveolar spaces, Masson trichrome staining of lung sections shows collagen fiber (blue). Black: nucleus; red: cytoplasm. Immunohistochemistry of collagen I **(B)** and fibronectin **(C)** expressions in bronchial epithelial and alveolar spaces (brown). Nucleus: blue. Red arrowhead: epithelial cells stain positive.

The effects of arsenic exposure on collagen fiber deposition in mice lung were determined by Masson trichrome staining. The collagen fiber deposition was significantly increased in the peri-bronchioles and alveolar region after medium (2mg/L) and high (50 mg/L) NaAsO_2_ exposure at 12 and 24 weeks. The low dose (0.2mg/L) of NaAsO_2_ exposure has no significant increase in collagen fiber deposition (**Figure 4A**, blue color). Furthermore, the medium- and high-dose NaAsO_2_ groups were applied to immunohistochemistry analysis using collagen I and fibronectin antibodies. In addition to the peri-bronchioles and alveolar region, the collagen I and fibronectin expressions were also upregulated in bronchial and alveolar epithelial cells (**Figures 4B, C**; red arrowhead). These results indicated that medium to high doses of arsenic exposure caused inflammation, structural changes, and collagen deposition in mice lung.

### 3.5 Arsenic induce mesenchymal changes of lung epithelial cell through ROS-MMP-2 activation

**Figure 5:**
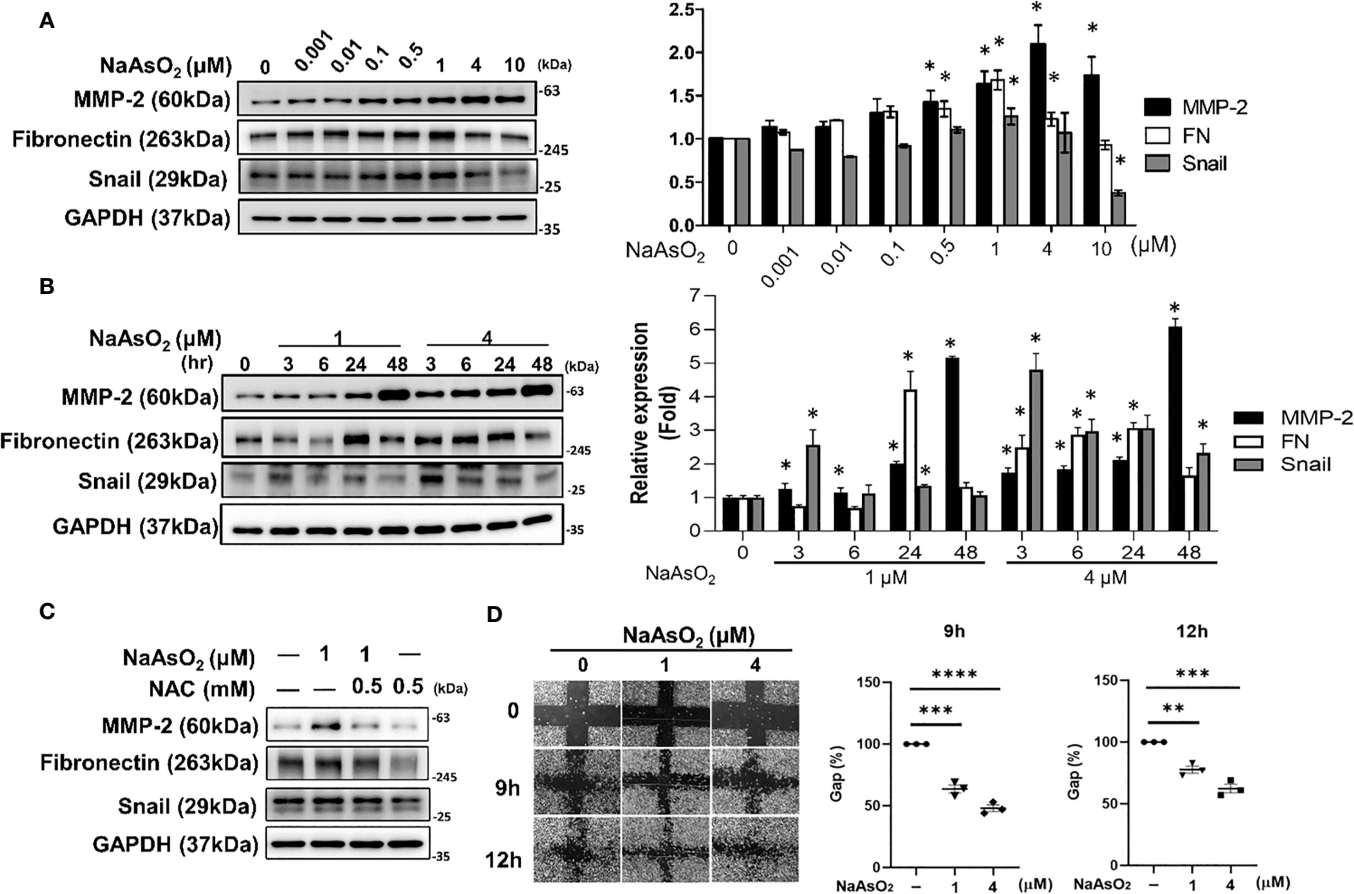
Effect of NaAsO_2_ on fibrinogenic protein expressions and cell migration of NHBE cells. **(A)** NHBE cells were treated with various doses of NaAsO_2_ from 10^-9^M to 10^-5^M for 24 hours. Western blot shows the expressions of fibrosis-related proteins. The quantitative results were shown in right panel. Data was expressed as Mean+/-SEM. *: *p*<0.05 compared with untreated control. **(B)** NHBE cells were treated with NaAsO_2_ 1μM and 4μM for 3, 6, 24, and 48 hours. The protein expressions of MMP-2, fibronectin, and Sanil were analyzed by western blot. The quantitative results were shown in right panel. Data was expressed as Mean+/-SEM. *: *p*<0.05 compared with untreated control. **(C)** NHBE cells were treated with NAC 2 hours before NaAsO_2_ treatment followed by combine treatment with NaAsO_2_ for 24 hours. The protein expression was analyzed by western blot. **(D)** NHBE cells were treated with 1μM and 4μM NaAsO_2_ for 24 hours, and the wound were made. The migration ability was determined by measure the area of the wound area at 9h and 12 h after wound made. Each result was performed in three independent experiments. Data was representative of three independent experiments and expressed as Mean+/-SEM. **: *p*<0.01 compared with untreated control, ***: *p*<0.001 compared with untreated control, ****: *p*<0.001 compared with untreated control.

Normal human bronchial epithelial (NHBE) cells were treated with various doses of NaAsO_2_ from 10^-9^ to 10^-3^ M for 24h. The expressions of matrix metalloproteinase-2 (MMP-2) Fibronectin (FN), and Snail were significantly induced by NaAsO_2_ treatment (**Figure 5A**). The timely effect of NaAsO_2_1uM and 4uM on MMP-2, Fibronectin, and Snail expressions were shown in **Figure 5B**. The results indicated that NaAsO_2_ induced time-and dose-dependent expression of MMP-2. The induction of fibronectin was dose-dependent and decreased after 48 hours of treatment. The induction of Snail was dramatic at 3h and this increase was attenuated by time. Furthermore, the NaAsO_2_-induced of MMP-2, Fibronectin, and Snail were reduced by pretreatment of N-acetyl cysteine (NAC) (**Figure 5C**). This result indicated the induction of mesenchymal marker expression by NaAsO_2_ was dependent on ROS generation. Finally, cell migration was enhanced by NaAsO_2_ at 1uM and 4uM as examined by wound healing assay (**Figure 5D**). Together these results demonstrated that arsenic treatment induced the mesenchymal phenotype which was mediated by ROS generation.

**Figure 6:**
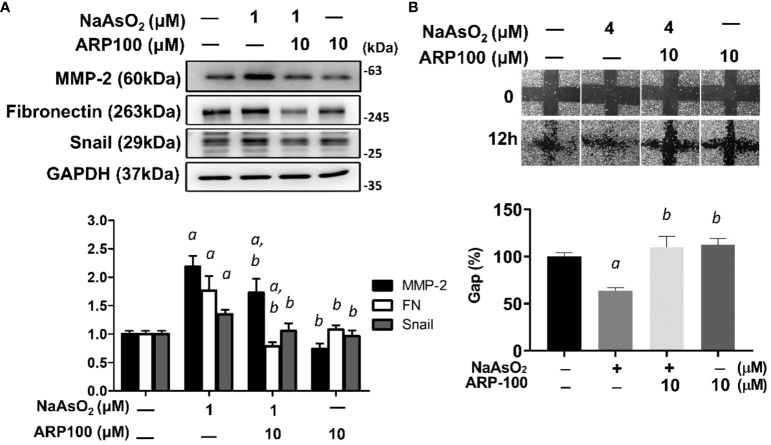
MMP-2 is critical for arsenic-induced EMT changes. **(A)** NHBE cells were pretreated with ARP100 2 hours before NaAsO_2_ treatment. After another 24 hours of combined treatment, the cells were applied for western blot analysis and wound healing assay. ARP-100 reverse NaAsO_2_-induced mesenchymal marker expressions **(A)** and cell migration **(B)**. Each result was performed in three independent experiments. The data were expressed as Mean+/-SEM. a: *p*<0.05 compared to untreated control; b: *p*<0.05 compared to NaAsO_2_ group.

The effects of MMP-2 inhibition on arsenic-induced mesenchymal changes of lung epithelial cells were examined. By using a selective inhibitor for MMP-2, ARP-100, the arsenic-induced fibronectin and Snail expressions were attenuated (**Figure 6A**). Simultaneously, the arsenic-induced cell migration was also significantly inhibited by ARP-100 (**Figure 6B**). These results indicated that MMP-2 is critical for arsenic-induced EMT changes.

### 3.6 Apigenin reversed NaAsO_2_-induced fibrogenic changes in vitro and in vivo

**Figure 7:**
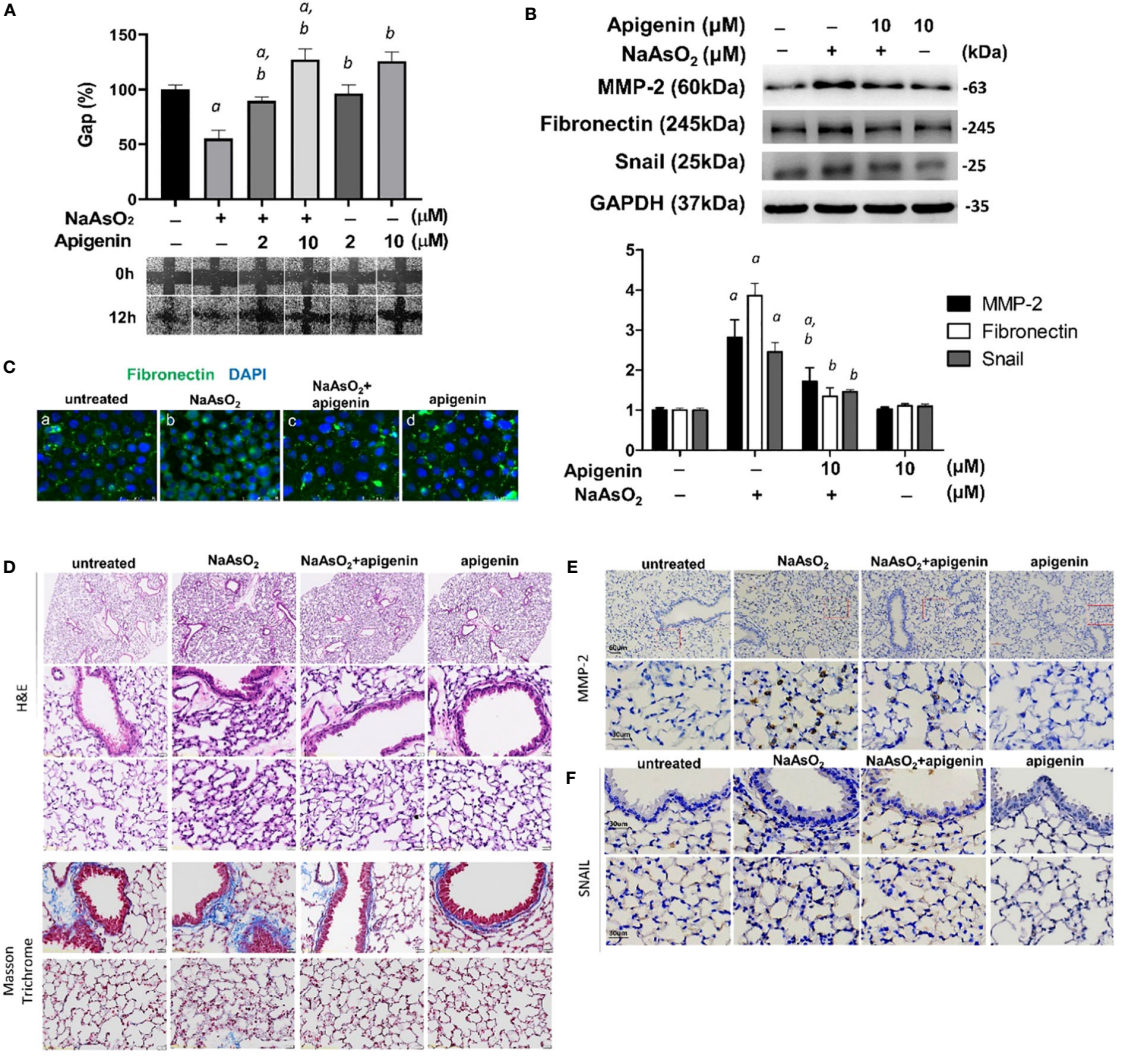
Apigenin reversed the NaAsO_2_-induced mesenchymal cell markers expressions, cell migration, and lung fibrosis of mice. **(A)** NHBE cells were pretreated with 2µM and 10µM apigenin for 2 hours followed by combined treatment with 4µM NaAsO_2_ for another 24 hours. The wound was made and the wound area at 0h and 12h after wound made were analyzed and expressed as Gap%. The representative images were shown. The data were expressed as Mean+/-SEM. All experiments were performed three times. a: statistical significance compared with control group; b: statistical significance compared with 4µM NaAsO_2_ group. **(B)** NHBE cells were treated with apigenin 2 hours prior 1µM NaAsO_2_ stimulation. After 24 hours of combined treatment, the cells were harvest for protein extraction and western blot analysis using antibodies as indicated. a: statistical significance compared with control group; b: statistical significance compared with NaAsO_2_ group. **(C)** NHBE cells were treated with 10µM of apigenin for 2 hours followed by combined treatment with 1µM NaAsO_2_ for additional 24 hours. The cells were fixed and applied for immunofluorescence against fibronectin. Green: fibronectin, blue: DAPI. Apigenin reversed the NaAsO_2_-induced histopathological changes and mesenchymal markers in mice lung. C57BL/6 mice at 6–8 weeks of age were treated with 50 mg/L NaAsO_2_ in the drinking water daily for 12 weeks with or without combined treatment with apigenin. **(D)** H&E stain, and Masson Trichrome stain, and immunohistochemistry against **(E)** MMP-2 and **(F)** Snail were shown.

NHBE cells were pretreated with apigenin and followed by combined treatment with NaAsO_2_ for 24hrs. Pretreatment of apigenin inhibited arsenic-induced cell migration (**Figure 7A**) and mesenchymal protein markers including MMP-2, fibronectin, and Snail (**Figure 7B**). Immunofluorescence images show the upregulated Fibronectin expression with aberrant localization by 1μM NaAsO_2_ treatment. which was ameliorated by apigenin in NHBE cells (**Figure 7C**). Mice were exposed to 50mg/L NaAsO_2_ through drinking water with or without daily administration of 10mg/kg body weight/day of apigenin for 12 weeks. Histology showed consistent pulmonary damage by 50mg/L NaAsO_2_ that caused an increase in alveolar septal thickness and cellularity. These effects were attenuated by combined treatment with apigenin (NaAsO_2_+apigenin group) (**Figures 7D**, H&E stain). Masson trichrome stain showed increased deposition of collagen fibers at the peri-bronchioles and alveolar region in the NaAsO_2_-only group, which were decreased in the NaAsO_2_+apigenin group (**Figure 7D**, Masson Trichrome). The apigenin-only group show no significant difference compared with the control group. The lung expressions of MMP-2 and Snail were upregulated by NaAsO_2_. MMP-2 expression was mainly detected in the lung parenchyma (**Figure 7E**), and Snail was detected in the nucleus of both bronchial and mesenchymal-epithelial cells (**Figure 7F**). The NaAsO_2_-induced upregulation of MMP-2 and Snail was significantly reduced by apigenin. These results indicated that apigenin is effective in ameliorating arsenic-induced pulmonary damage, and the expressions of MMP-2 and Snail are correlated with arsenic-induced pulmonary damage.

## 4 Discussion

Our study is the first to reveal that exposure to high levels of arsenic is associated with an elevated risk of lung fibrotic changes. Additionally, we found that changes in lung function tests were affected by changes in arsenic levels. *In vitro*, sodium arsenite treatment promotes the epithelial-mesenchymal transition (EMT)-like changes of the normal human bronchial epithelial cells, including upregulation of several fibrotic and mesenchymal markers (fibronectin, MMP-2, and Snail) and cell migration. Inhibition of reactive oxygen species (ROS) and MMP-2 impaired the arsenic-induced EMT changes. Administration of a flavonoid, apigenin, inhibited EMT *in vitro* and pulmonary damages *in vivo* with the reduction of mesenchymal markers. Collectively, these results suggested that EMT is a critical initial event for arsenic-induced lung fibrotic changes.

Our analysis revealed that there was a significant difference in the slope of lung function changes between As^LtoH^ and As^HtoL^ groups compared to the As^LtoL^ group. However, there was no significant difference in the slope changes of lung function between the As^HtoH^ and As^LtoL^ groups (**Figure 1**). Although there are some studies that have shown an association between changes in urinary arsenic levels and lung function tests, the evidence is limited. Previous studies have suggested that higher levels of urinary arsenic are linked to a decline in lung function tests (5), but to date, no studies have demonstrated that decreasing arsenic exposure can improve or increase lung function tests. Our study indicates that decreasing urinary arsenic exposure may prevent the deterioration of lung function. Additionally, our data suggest that lung function tests can recover to baseline levels, similar to the population with low levels of arsenic exposure, within a few years (in our study, within 2 years). We recommend reducing arsenic exposure to prevent declines in lung function and our findings provide evidence that lung function tests can improve after a decrease in arsenic exposure.

In our research, we have defined the diagnosis of lung fibrotic changes on LDCT images as the presence of curvilinear or linear densities, fine lines, or plate opacity in specific lobes. It is important to note that the term “lung fibrotic changes” is distinct from “lung fibrosis”. We specifically use the term idiopathic pulmonary fibrosis (IPF) to refer to a chronic and progressive fibrosing interstitial pneumonia that primarily affects older adults and is restricted to the lungs. The histopathological pattern of IPF is known as usual interstitial pneumonia (UIP), and a definite diagnosis of UIP on LDCT requires the presence of honeycombing, a distinguishing feature. The typical distribution of UIP is subpleural with basal predominance, but upper lobe involvement is also common. LDCT findings of reticular pattern with peripheral traction bronchiectasis or bronchiectasis are considered probable UIP. The main diagnostic feature and characteristic histopathologic finding of UIP is the presence of patchy, dense fibrosis observed at low magnification, which leads to remodeling of the lung architecture, often resulting in the development of honeycomb changes. While lung fibrotic changes can occur as a consequence of lung inflammation or infection (32), there are currently no globally recognized criteria or grading systems to classify them, and it is unclear whether and how these changes overlap with lung fibrosis. The causes of pulmonary fibrosis include cigarette smoking, exposure to environmental risk factors such as particulate matter inhalation, and exposure to metal, asbestos, and wood dust, as well as connective tissue disease and drug use. Some studies have suggested a potential association between heavy metal exposure and the increasing incidence of IPF in older patients, but further research is needed to investigate the correlation between arsenic exposure and lung fibrosis. It remains uncertain whether the rise in the occurrence of pulmonary fibrosis is due to idiopathic factors or a surge in secondary factors that lead to lung fibrosis. Our studies have shed light on the correlation between arsenic exposure and lung fibrotic changes, providing evidence that environmental exposure, such as exposure to arsenic, may be a secondary cause of lung fibrosis.

Mice exposed to 2mg/L NaAsO_2_ (equal to 1.2 ppm of arsenic) for 12 weeks show significant structural changes of the lung by histology analysis (**Figure 3**). For drug dosage and age translation from mice to humans (33), that was equal to approximately 0.1 ppm of arsenic (10-fold higher than the standard for arsenic in drinking water) exposure for 9.2 years for humans. We also performed 6 weeks of exposure to high-dose arsenic (equal to 2.4 ppm exposure for 4.6 years in humans), and the results show no significant differences in lung histology by histological analysis.

More recently, several studies have demonstrated that arsenic exposure through drinking water would lead to lung fibrosis in rodents (16–18). Lung fibrotic changes, including the, destroy of the alveolar wall, inflammatory cell infiltration, and collagen deposition, could be detected earliest by 4 months of exposure with 2.5~10mg/kg/day of NaAsO_2_ (equals to 1.5~6 ppm of arsenic) (16). In another study, mice exposed to a relatively low dose of NaAsO_2_ (5 ppm) for 6 months would lead to narrow alveolar space and the upregulation of fibrotic markers (COL1A2 and α-SMA) (17). Moreover, the lung fibrosis phenomenon including reticular alternations, irregular septal thickening, and increased tissue density of the lung was observed in mice exposed to 10~20 ppm for 12 months (18). Consistent with these studies, we found the thickened alveolar septum after 3 months of arsenic exposure (28.8ppm, 50mg) and accompanied by the upregulation of MMP-2 and Snail. For 6 months of arsenic treatment, collagen fiber deposition was significantly increased at the peri-bronchioles and alveolar region. For bronchial, it showed a characteristic of airway remodeling, which has a thickened epithelial layer and subepithelial basement membrane layer (**Figure 3**). For the alveoli, aberrant and narrowed alveolar space due to deposition of collagen fiber and infiltrated immune cells. This phenomenon suggested that arsenic exposure may affect both obstructive and restrictive pulmonary diseases.

On the other hand, our results from the human study showed that As^LtoH^ in a 2-year interval significantly correlated with the incidence of lung fibrotic changes (**Table 3**) and decreased lung function (**Figure 1**). The differences may be caused by the methods for fibrosis detection (human vs mice, LDCT vs histology). Application of micro-CT on mice fibrosis analysis was demonstrated to have a good correlation with histological evaluation (34, 35). Longitudinal measurements of lung fibrosis in mice using micro-CT may provide advanced evidence on arsenic-induced lung fibrosis in mice. Besides, the people recruited in this study were mostly residents of the industry area that may have prior exposure history as well as the lower urinary Arsenic groups at the first measurement in 2016 (As^LtoH^ and As^LtoL^). The high background arsenic exposure may affect further exposure time to cause fibrosis.

The epithelial cell is the abundant cell type of the lung and is considered critical for the initiation and progression of fibrosis (36). Here we demonstrated that the exposure of the environmentally relevant concentration of NaAsO_2_ (10^-6^M equals 75 ppb) induced EMT of lung epithelial cells *in vitro*. The migration activity was increased accompanied by a significantly increasing in mesenchymal protein expressions. MMP-2 and Fibronectin were categorized as type I (developmental), and type II (fibrosis and wound healing) specific EMT markers, but not the type III EMT marker (cancer) (37), indicating that arsenic activates the fibrogenic-type EMT.

EMT of pulmonary epithelial cells increases the number of fibroblasts, thus promoting the progression of pulmonary fibrosis. It is supported by the previous study which indicated alveolar cells were progenitors for fibroblasts (38). Through the establishment of lung-epithelial specific <υ>β</υ>-gal reporter mice, double positive cells expressing <υ>β</υ>-gal and myofibroblast marker (<υ>α</υ>-SMA) demonstrated EMT *in situ* under TGF-<υ>β</υ>-inducing pulmonary fibrosis (38). Here we demonstrated that arsenic exposure can directly induce EMT of epithelial cells using *in vitro* model, which is considered to contribute to an increase in the number of fibroblasts during fibrosis. Moreover, we also demonstrate the expressions of EMT markers in the lung of arsenic-exposed mice (**Figure 7**), indicating EMT is involved in arsenic-induced lung fibrosis.

Moreover, the previous study shows that alveolar epithelial cells (AECs) exposed to provisional fibronectin are sufficient to drive EMT *in vitro*. On the other hand, AECs exposed to provisional laminin/collagen I mixture and TGF-<υ>β</υ>1 undergo apoptosis rather than EMT *in vitro* (38). In our study, it is demonstrated that the upregulation of both collagen I and FN by arsenic in mice lung. It is suggested the production of FN further promotes the EMT of lung epithelial cells. The upregulation of collagen I may be associated with arsenic-induced pulmonary epithelial apoptosis.

Furthermore, by using chemical inhibitors to block the specific pathway, we demonstrated the involvement of ROS-MMP-2 pathways in promoting pulmonary epithelial cell EMT. The cell model was then used as a platform for screening the possible therapeutic agents. Flavonoids, which include flavones, flavonols, flavanones, flavanols, isoflavonoids, and anthocyanidins, were known to have broad biological activities, such as anti-oxidant and anti-inflammation. Apigenin is the most widely distributed and most studied flavone that has effects on diabetes, cancer, Alzheimer’s disease, amnesia (19), and also fibrosis (20, 39) through modulation of the cell-signaling pathways such as PI3K-AKT-GSK3, JAK-STAT, and MAPK signaling (40). Here, apigenin shows prominent effects on inhibition of the arsenic-induced fibrotic changes both *in vitro* and *in vivo.* It is suggested that this effect was caused by, at least partly, the anti-oxidant and GSK3 inhibition activity of apigenin.

MMP-2 and MMP-9 are the predominant types of airway MMPs expressed by epithelial cells (41). In a bleomycin-induced lung fibrosis rabbit model, MMP-2 expression and activity were increased at 3, 7, 14, and 28 days after bleomycin treatment in type II alveolar epithelial cells (42). Moreover, MMP-2 was upregulated in a lipopolysaccharide-induced lung fibrosis model and was considered to cause damage to the lung extracellular matrix as well as be associated with the development of lung fibrosis (43). In this study, we have found that MMP-2 was upregulated by arsenic in lung epithelial cells *in vitro* and *in vivo*. Block MMP-2 activity reduced fibrogenic marker expressions, suggesting that MMP-2 plays a critical role in the activation of further pro-fibrogenic events.

The strength of the present study is that it applied a large-scale population-based design to investigate the association between urinary arsenic levels and lung abnormalities on chest LDCT images. Meanwhile, the limitation is that we could not completely adjust for potentially unmeasured confounders (other environmental and genetic factors). Moreover, the effects of chronic arsenic exposure on lung function changes in mice were not performed in this study. The previous study has shown that acute arsenic exposure through intratracheal instillation induced lung function decline in mice (44). Although the effects of arsenic exposure on fibrotic changes in mice lung were proved by histological analysis in this study, chronic arsenic exposure through drinking water needs further investigation.

To summarize, we performed a large-scale longitudinal cohort study, cell model, and mice model to study the effect of arsenic on lung toxicity. Arsenic causes lung function impairment and lung fibrosis in a dose- and time-dependent manner. In the cell model, we demonstrated that arsenic activated the EMT-related changes including fibrogenic marker expressions of lung epithelial cells. Mice exposed to arsenic show increased collagen deposition at both subepithelial basement membrane and perialveolar space, which cause narrowed airway and alveoli space. Apigenin or medications targeting ROS generation and GSK3-MMP2 signaling were potential therapeutic strategies for arsenic-induced pulmonary damage.

## Data Availability

The datasets generated for this article are not readily available because of ethical restrictions. Requests to access the datasets should be directed to Dr. C-WW (e-mail: 960405@kmuh.org.tw).
