# Corrigendum: Arsenic exposure and lung fibrotic changes-evidence from a longitudinal cohort study and experimental models

**DOI:** 10.3389/fimmu.2024.1473483

**Published:** 2024-11-12

**Authors:** Chih-Wen Wang, Hsin-Ying Clair Chiou, Szu-Chia Chen, Da-Wei Wu, Hung-Hsun Lin, Huang-Chi Chen, Wei-Ting Liao, Ming-Hong Lin, Chih-Hsing Hung, Chao-Hung Kuo

**Affiliations:** ^1^ Division of Hepatobiliary, Department of Internal Medicine, Kaohsiung Medical University Hospital, Kaohsiung Medical University, Kaohsiung, Taiwan; ^2^ Department of Internal Medicine, Kaohsiung Municipal Siaogang Hospital, Kaohsiung Medical University, Kaohsiung, Taiwan; ^3^ Teaching and Research Center, Kaohsiung Municipal Siaogang Hospital, Kaohsiung Medical University, Kaohsiung, Taiwan; ^4^ Kaohsiung Medical University Hospital, Kaohsiung Medical University, Kaohsiung, Taiwan; ^5^ Department of Applied Chemistry, National Chi Nan University, Nantou, Taiwan; ^6^ Division of Nephrology, Department of Internal Medicine, Kaohsiung Medical University Hospital, Kaohsiung Medical University, Kaohsiung, Taiwan; ^7^ Research Center for Environmental Medicine, Kaohsiung Medical University, Kaohsiung, Taiwan; ^8^ Faculty of Medicine, College of Medicine, Kaohsiung Medical University, Kaohsiung, Taiwan; ^9^ Division of Pulmonary and Critical Care Medicine, Department of Internal Medicine, Kaohsiung Medical University Hospital, Kaohsiung Medical University, Kaohsiung, Taiwan; ^10^ Department of Biotechnology, College of Life Science, Kaohsiung Medical University, Kaohsiung, Taiwan; ^11^ Department of Medical Research, Kaohsiung Medical University Hospital, Kaohsiung Medical University, Kaohsiung, Taiwan; ^12^ Department of Microbiology and Immunology, School of Medicine, College of Medicine, Kaohsiung Medical University, Kaohsiung, Taiwan; ^13^ M.Sc. Program in Tropical Medicine, College of Medicine, Kaohsiung Medical University, Kaohsiung, Taiwan; ^14^ Department of Pediatrics, Kaohsiung Medical University Hospital, Kaohsiung Medical University, Kaohsiung, Taiwan; ^15^ Department of Pediatrics, Kaohsiung Municipal Siaogang Hospital, Kaohsiung Medical University, Kaohsiung, Taiwan; ^16^ Division of Gastroenterology, Department of Internal Medicine, Kaohsiung Medical University Hospital, Kaohsiung Medical University, Kaohsiung, Taiwan

**Keywords:** arsenic, lung fibrosis, epithelial-mesenchymal transition, apigenin, LDCT images

In the published article, there was an error in the **Data Availability statement**. “The raw data supporting the conclusions of this article will be made available by the authors, without undue reservation.” The correct **Data Availability statement** appears below.

“The datasets generated for this article are not readily available because of ethical restrictions. Requests to access the datasets should be directed to Dr. Chih-Wen Wang (e-mail: 960405@kmuh.org.tw).”

Furthermore in the published article, there was an error in **Figure 6B**. The concentration of NaAsO_2_ should be labeled as 4 μM. The corrected **Figure 6** appears below.

**Figure 6:**
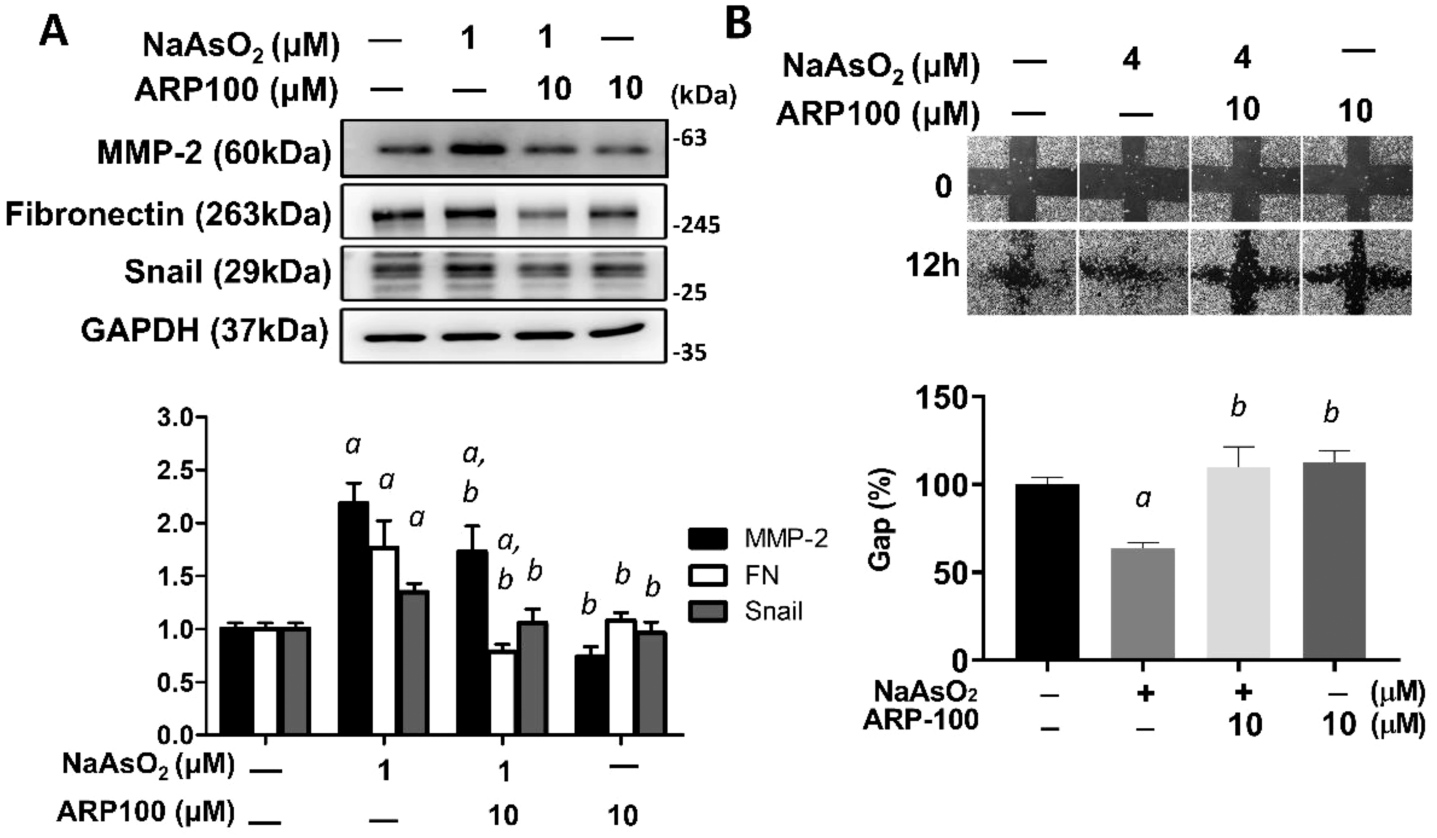
MMP-2 is critical for arsenic-induced EMT changes. **(A)** NHBE cells were pretreated with ARP100 2 hours before NaAsO_2_ treatment. After another 24 hours of combined treatment, the cells were applied for western blot analysis and wound healing assay. ARP-100 reverse NaAsO_2_-induced mesenchymal marker expressions **(A)** and cell migration **(B)**. Each result was performed in three independent experiments. The data were expressed as Mean+/-SEM. a: *p*<0.05 compared to untreated control; b: *p*<0.05 compared to NaAsO_2_ group.

There was also an error in the legend for **Figure 7** for NaAsO_2_ concentration as published. The concentrations of NaAsO_2_ used in **Figures 7A** and **7B** experiments were 4μM and 1μM respectively which are described in more detail in figure legend.

**Figure 7:**
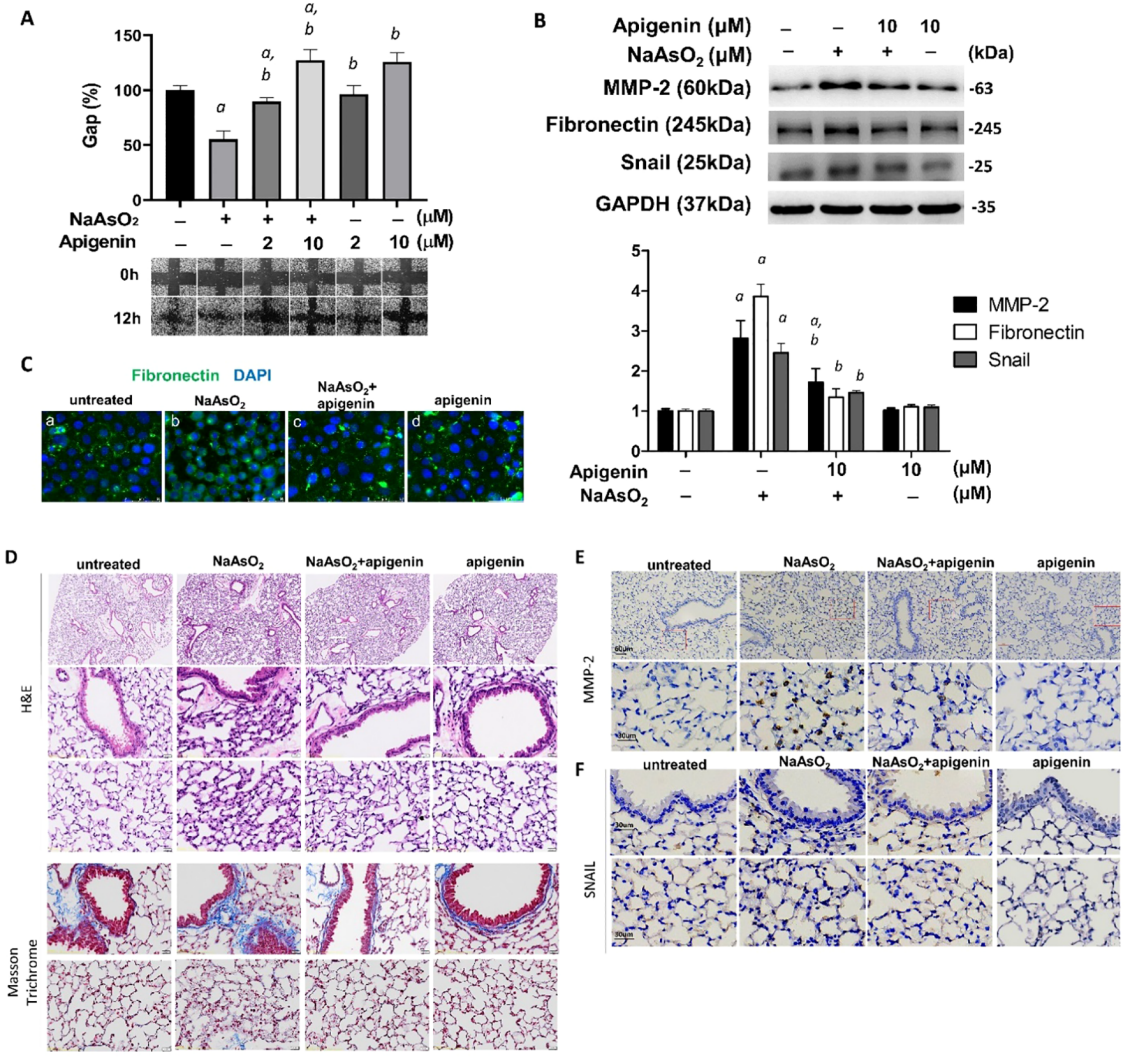
Apigenin reversed the NaAsO_2_-induced mesenchymal cell markers expressions, cell migration, and lung fibrosis of mice. **(A)** NHBE cells were pretreated with 2μM and10μM apigenin for 2 hours followed by combined treatment with 4μM NaAsO_2_ for another 24 hours. The wound was made and the wound area at 0h and 12h after wound made were analyzed and expressed as Gap%. The representative images were shown. The data were expressed as Mean+/-SEM. All experiments were performed three times. a: statistical significance compared with control group; b: statistical significance compared with 4μM NaAsO_2_ group. **(B)** NHBE cells were treated with apigenin 2 hours prior 1μM NaAsO_2_ stimulation. After 24 hours of combined treatment, the cells were harvest for protein extraction and western blot analysis using antibodies as indicated. a: statistical significance compared with control group; b: statistical significance compared with NaAsO_2_ group. **(C)** NHBE cells were treated with 10μM of apigenin for 2 hours followed by combined treatment with 1μM NaAsO_2_ for additional 24 hours. The cells were fixed and applied for immunofluorescence against fibronectin. Green: fibronectin, blue: DAPI. Apigenin reversed the NaAsO_2_-induced histopathological changes and mesenchymal markers in mice lung. C57BL/6 mice at 6–8 weeks of age were treated with 50 mg/L NaAsO_2_ in the drinking water daily for 12 weeks with or without combined treatment with apigenin. **(D)** H&E stain, and Masson Trichrome stain, and immunohistochemistry against **(E)** MMP-2 and **(F)** Snail were shown.

“(A) NHBE cells were pretreated with 2μM and10μM apigenin for 2 hours followed by combined treatment with 1μM NaAsO_2_ for another 24 hours. The wound was made and the wound area at 0h and 12h after wound made were analyzed and expressed as Gap%. The representative images were shown. The data were expressed as Mean+/-SEM. All experiments were performed three times. *a*: statistical significance compared with control group; *b*: statistical significance compared with 1μM NaAsO_2_ group.” The corrected legend appears below.

“(A) NHBE cells were pretreated with 2μM and10μM apigenin for 2 hours followed by combined treatment with 4μM NaAsO_2_ for another 24 hours. The wound was made and the wound area at 0h and 12h after wound made were analyzed and expressed as Gap%. The representative images were shown. The data were expressed as Mean+/-SEM. All experiments were performed three times. *a*: statistical significance compared with control group; *b*: statistical significance compared with 4μM NaAsO_2_ group.”

In the published article, there was an error in **Figure 7F** as published. The image of “apigenin” group is carelessly misplaced during figure organization and caused the image duplication. The corrected **Figure 7** appear below.

Lastly, a correction has been made to **Section 3.6 Apigenin reversed NaAsO_2_-induced Fibrogenic changes *in vitro* and *in vivo***, Paragraph Number 1. This sentence previously stated:

“NHBE cells were pretreated with apigenin and followed by combined treatment with 1μM NaAsO_2_ for 24hrs.”

The corrected sentence appears below:

“NHBE cells were pretreated with apigenin and followed by combined treatment with NaAsO_2_ for 24hrs.”

The authors apologize for these errors and state that this does not change the scientific conclusions of the article in any way.

