# Supplementary material for: Arsenic exposure and lung fibrotic changes-evidence from a longitudinal cohort study and experimental models
Source: Front Immunol. 2023 Aug 22;14:1225348. doi: 10.3389/fimmu.2023.1225348 (PMC10477983; doi:10.3389/fimmu.2023.1225348)
Supplement: Supplementary file 1 [file DataSheet1.doc]

**Supplementary materials**

**Arsenic exposure and pulmonary fibrosis-evidence from a longitudinal cohort study and experimental models**

Chih-Wen Wang^1,2^, Hsin-Ying Clair Chiou^3,4,5#^, Szu-Chia Chen^2,6,7,8^, Da-Wei Wu^2,9^, Hung-Hsun Lin^10^, Huang‐Chi Chen^2,9^, Wei-Ting Liao^7,11, 12^, Ming-Hong Lin^12,13,14**^, Chih-Hsing Hung^7,15,16*^, Chao-Hung Kuo^2,8, 17**^

**Supplement Table of content**

**Table S1** Demographic characteristics and urinary arsenic levels by lung fibrotic changes in 2016 and 2018 (n= 976)

**Table S2** Urinary arsenic levels and lung function tests in all participants (n= 976)

**Table S3** Multiple linear regression analysis for urinary arsenic levels associated with lung function tests (n= 976)

**Table S4** Mean difference of lung function tests associated with changes of urinary arsenic levels by time (2016-2018) (n= 976)

Table S1 Demographic characteristics and urinary arsenic levels by lung fibrotic changes in 2016 and 2018 (n= 976)

| Characteristics | Lung fibrotic^*^ | Lung fibrotic^*^ | Lung fibrotic^*^ | Lung fibrotic^*^ | P |
| --- | --- | --- | --- | --- | --- |
|  | Negative to Negative | Positive to Negative | Negative to Positive | Positive to Positive |  |
|  | (n=394) | (n=167) | (n=159) | (n=256) |  |
| Continuous variable, mean (SD) | | | | | |
| Age (yrs.) | 54.8(10.7) | 58(11.3) | 55.6(9.6) | 61.6(10.6) | <0.001 |
| BMI (Kg/m^2^) | 25.3(3.9) | 24.9(4.5) | 24.7(4.8) | 24.7(3.3) | 0.386 |
| Geometric mean of urinary arsenic levels (μg/g creatinine) | | | | | |
| Arsenic (visit 1,2016) | 86.0 | 90.9 | 81.7 | 110.1 | 0.001 |
| Arsenic (visit 2,2018) | 80.9 | 91.2 | 89.8 | 94.9 | 0.040 |
| Category variable, n (%) | | | | | |
| Gender |  |  |  |  | 0.779 |
| Female | 346(62.3) | 78(67.2) | 43(65.2) | 151(63.2) |  |
| Male | 209(37.7) | 38(32.8) | 23(34.8) | 88(36.8) |  |
| Educational level |  |  |  |  | 0.001 |
| ≤junior high school | 248(44.7) | 58(50.0) | 29(43.9) | 146(61.1) |  |
| Senior high school | 191(34.4) | 44(37.9) | 23(34.8) | 65(27.2) |  |
| ≥college | 116(20.9) | 14(12.1) | 14(21.2) | 28(11.7) |  |
| Smoking |  |  |  |  | 0.966 |
| Yes | 59(10.6) | 13(11.2) | 7(10.6) | 23(9.6) |  |
| No | 496(89.4) | 103(88.8) | 59(89.4) | 216(90.4) |  |
| Alcohol consumption |  |  |  |  | 0.166 |
| Yes | 107(19.3) | 15(12.9) | 14(21.2) | 46(19.2) |  |
| No | 448(80.7) | 101(87.1) | 52(78.8) | 193(80.8) |  |
| Betel chewing |  |  |  |  | 0.723 |
| Yes | 13(2.3) | 1(0.9) | 1(1.5) | 4(1.7) |  |
| No | 542(97.7) | 115(99.1) | 65(98.5) | 235(98.3) |  |
| Air purifier |  |  |  |  | 0.060 |
| Yes | 449(80.9) | 99(85.3) | 59(89.4) | 209(87.4) |  |
| No | 106(19.1) | 17(14.7) | 7(10.6) | 30(12.6) |  |
| Diabetes mellitus |  |  |  |  | 0.313 |
| Yes | 175(31.5) | 33(28.4) | 21(31.8) | 89(37.2) |  |
| No | 380(68.5) | 83(71.6) | 45(68.2) | 150(62.8) |  |
| Hypertension |  |  |  |  | 0.068 |
| Yes | 321(57.8) | 68(58.6) | 40(60.6) | 162(67.8) |  |
| No | 234(42.2) | 48(41.4) | 26(39.4) | 77(32.2) |  |

Pairwise lung fibrotic changes: "Lung fibrotic _negative to negative_" indicating absence of lung fibrotic changes in both 2016 and 2018,"Lung fibrotic _negative to positive_" indicating no lung fibrotic changes in 2016 but present in 2018, "Lung fibrotic _positive to negative_" indicating lung fibrotic changes in 2016 but not in 2018, and "Lung fibrotic _positive to positive_" indicating lung fibrotic changes present in both 2016 and 2018

Table S2 Distribution of total urinary arsenic levels and lung function tests in all participants (n= 976)

| Items | Mean | SD | GM | Min. | 25th | Median | 75th | Max. |
| --- | --- | --- | --- | --- | --- | --- | --- | --- |
| 2016 |  |  |  |  |  |  |  |  |
| Arsenic | 137.41 | 163.03 | 91.67 | 3.60 | 50.75 | 86.95 | 154.15 | 1434.90 |
| FEV1 (L) | 2.33 | 0.68 | 2.23 | 0.52 | 1.83 | 2.24 | 2.76 | 4.53 |
| FVC (L) | 2.58 | 0.77 | 2.46 | 0.61 | 2.01 | 2.47 | 3.04 | 5.29 |
| FEV1/FVC | 90.91 | 7.50 | 90.57 | 41.74 | 86.66 | 91.65 | 97.93 | 99.96 |
| 2018 |  |  |  |  |  |  |  |  |
| Arsenic | 122.20 | 125.80 | 86.91 | 8.90 | 45.73 | 85.05 | 151.23 | 1548.30 |
| FEV1 (L) | 2.28 | 0.71 | 2.17 | 0.53 | 1.75 | 2.17 | 2.73 | 5.61 |
| FVC (L) | 2.45 | 0.76 | 2.33 | 0.55 | 1.90 | 2.32 | 2.92 | 6.19 |
| FEV1/FVC | 93.10 | 6.48 | 92.89 | 60.22 | 89.25 | 94.70 | 98.68 | 100.00 |

Arsenic (μg/g creatinine)

Table S3 Multiple linear regression analysis for urinary arsenic levels associated with lung function tests (n= 976)

| Items | β | 95% CI | P |
| --- | --- | --- | --- |
| Visit 1 (2016) |  |  |  |
| FVC |  |  |  |
| Crude | -0.438 | (-0.565,-0.311) | <0.001 |
| Model 1 | -0.190 | (-0.281,-0.098) | <0.001 |
| Model2 | -0.119 | (-0.210,-0.029) | 0.010 |
| FEV1 |  |  |  |
| Crude | -0.410 | (-0.523,-0.298) | <0.001 |
| Model 1 | -0.178 | (-0.258,-0.097) | <0.001 |
| Model2 | -0.111 | (-0.190,-0.033) | 0.006 |
|  |  |  |  |
| Visit 2 (2018) |  |  |  |
| FVC |  |  |  |
| Crude | -0.415 | (-0.546,-0.284) | <0.001 |
| Model 1 | -0.206 | (-0.304,-0.107) | <0.001 |
| Model2 | -0.118 | (-0.216,-0.020) | 0.018 |
| FEV1 |  |  |  |
| Crude | -0.408 | (-0.531,-0.286) | <0.001 |
| Model 1 | -0.203 | (-0.295,-0.112) | <0.001 |
| Model2 | -0.120 | (-0.211,-0.029) | 0.010 |

Model 1 adjusted for age, gender, and BMI, Model 2 adjusted for age, gender, BMI, smoking, education, air purifier, hypertension, and diabetes mellitus

Table S4 Mean difference of lung function tests associated with changes of urinary arsenic levels by time (2016-2018) (n= 976)

| Arsenic changes | | FVC | | | | FEV1 | | | |
| --- | --- | --- | --- | --- | --- | --- | --- | --- | --- |
|  |  | Mean  difference | 95% CI | | p | Mean  difference | 95% CI | | p |
| Visit 2 (2018)  vs.  Visit 1 (2016) | As^HtoH^ | -0.09 | (-0.14,-0.04) | | 0.001 | -0.02 | (-0.06,0.02) | | 0.353 |
|  | As^LtoH^ | -0.20 | (-0.26,-0.14) | | <0.001 | -0.11 | (-0.16,-0.05) | | <0.001 |
|  | As^HtoL^ | -0.01 | (-0.07,0.06) | | 0.848 | 0.04 | (-0.01,0.09) | | 0.122 |
|  | As^LtoL^ | -0.11 | (-0.15,-0.06) | | <0.001 | -0.03 | (-0.07,0.002) | | 0.063 |
| Visit 1 (2016) | | | | | | | | | |
| As^LtoL^ vs. | As^HtoH^ | 0.11 | (0.02,0.19) | | 0.015 | 0.11 | (0.03,0.18) | | 0.006 |
|  | As^LtoH^ | 0.04 | (-0.06,0.14) | | 0.402 | 0.05 | (-0.03,0.14) | | 0.213 |
|  | As^HtoL^ | 0.15 | (0.05,0.24) | | 0.003 | 0.14 | (0.05,0.22) | | 0.001 |
| As^HtoL^ vs. | As^HtoH^ | -0.04 | (-0.14,0.06) | | 0.438 | -0.03 | (-0.12,0.06) | | 0.505 |
|  | As^LtoH^ | -0.11 | (-0.22,0.01) | | 0.067 | -0.08 | (-0.18,0.02) | | 0.100 |
| As^LtoH^ vs. | As^HtoH^ | 0.07 | (-0.04,0.17) | | 0.217 | 0.05 | (-0.04,0.14) | | 0.256 |
|  |  |  |  |  |  |  |  |  |  |
| Visit 2 (2018) | | | | | | | | | |
| As^LtoL^ vs. | As^HtoH^ | 0.09 | (0.002,0.18) | | 0.046 | 0.09 | (0.01,0.17) | | 0.024 |
|  | As^LtoH^ | 0.13 | (0.04,0.23) | | 0.008 | 0.13 | (0.04,0.22) | | 0.006 |
|  | As^HtoL^ | 0.05 | (-0.05,0.14) | | 0.349 | 0.06 | (-0.03,0.15) | | 0.174 |
| As^HtoL^ vs. | As^HtoH^ | 0.04 | (-0.06,0.15) | | 0.424 | 0.03 | (-0.07,0.13) | | 0.541 |
|  | As^LtoH^ | 0.09 | (-0.03,0.20) | | 0.136 | 0.07 | (-0.04,0.17) | | 0.230 |
| As^LtoH^ vs. | As^HtoH^ | 0.05 | (-0.15,0.06) | | 0.399 | -0.04 | (-0.13,0.06) | | 0.478 |

Adjusted for age, gender, BMI, smoking, education levels, air purifier use, hypertension, and diabetes mellitus, Abbreviation: As, arsenic
