# Supplementary material for: Arsenic exposure and lung fibrotic changes-evidence from a longitudinal cohort study and experimental models
Source: Front Immunol. 2023 Aug 22;14:1225348. doi: 10.3389/fimmu.2023.1225348 (PMC10477983; doi:10.3389/fimmu.2023.1225348)
Supplement: Supplementary file 2 [file DataSheet2.doc]

**Population living near a petrochemical complex health Survey**

**Southern Taiwan**

(Age: 39 – 91 years old)

**Visit 1 (2016), n= 989**

1. Self-reported questionnaires and anthropometric measurements (body weight, height, smoking, air purifier use, and lung carcinoma history)
2. Urinary arsenic levels
3. Chest low-dose computed tomography (LDCT)
4. Spirometry forced expiratory volume in 1 s (FEV1), forced vital capacity (FVC)

**Excluding criteria**: Lung carcinoma (n= 8), pulmonary tuberculosis (n=1), asthma history (n=4)

**Visit 2 (2018), n= 989**

1. LDCT performed for all participants
2. Urinary arsenic levels
3. Spirometry FEV1, FVC

**Final participants into analysis: n= 976**

1. Pairwise urinary Arsenic changes: As^LtoL^, As^HtoL^, As^LtoH^ and As^HtoH^
2. Pairwise lung fibrotic changes: Lung fibrotic _negative to negative_, Lung fibrotic _positive to negative_, Lung fibrotic _negative to positive_ and Lung fibrotic _positive to positive_

Figure S1: Flow-chart of the study design
